# Custom 4-Plex DiLeu Isobaric Labels Enable Relative Quantification of Urinary Proteins in Men with Lower Urinary Tract Symptoms (LUTS)

**DOI:** 10.1371/journal.pone.0135415

**Published:** 2015-08-12

**Authors:** Tyler Greer, Ling Hao, Anatoliy Nechyporenko, Sanghee Lee, Chad M. Vezina, Will A. Ricke, Paul C. Marker, Dale E. Bjorling, Wade Bushman, Lingjun Li

**Affiliations:** 1 Department of Chemistry, University of Wisconsin-Madison, Madison, Wisconsin, United States of America; 2 School of Pharmacy, University of Wisconsin-Madison, Madison, Wisconsin, United States of America; 3 Department of Urology, University of Wisconsin-Madison, Madison, Wisconsin, United States of America; 4 School of Veterinary Medicine, University of Wisconsin-Madison, Madison, Wisconsin, United States of America; Moffitt Cancer Center, UNITED STATES

## Abstract

The relative quantification of proteins using liquid chromatography mass spectrometry (LC-MS) has allowed researchers to compile lists of potential disease markers. These complex quantitative workflows often include isobaric labeling of enzymatically-produced peptides to analyze their relative abundances across multiple samples in a single LC-MS run. Recent efforts by our lab have provided scientists with cost-effective alternatives to expensive commercial labels. Although the quantitative performance of these dimethyl leucine (DiLeu) labels has been reported using known ratios of complex protein and peptide standards, their potential in large-scale proteomics studies using a clinically relevant system has never been investigated. Our work rectifies this oversight by implementing 4-plex DiLeu to quantify proteins in the urine of aging human males who suffer from lower urinary tract symptoms (LUTS). Protein abundances in 25 LUTS and 15 control patients were compared, revealing that of the 836 proteins quantified, 50 were found to be differentially expressed (>20% change) and statistically significant (p-value <0.05). Gene ontology (GO) analysis of the differentiated proteins showed that many were involved in inflammatory responses and implicated in fibrosis. While confirmation of individual protein abundance changes would be required to verify protein expression, this study represents the first report using the custom isobaric label, 4-plex DiLeu, to quantify protein abundances in a clinically relevant system.

## Introduction

Liquid chromatography mass spectrometry (LC-MS) is frequently utilized to quantify proteins by comparing the relative abundances of enzymatically produced peptides in disease versus control states [1–4]. Quantitative MS approaches are either label-free [5–9] or label-based [10–15]. Labeling methods allow multiplexing of samples and enable comparative analysis in a single LC-MS run. Thus, labeling can increase throughput, improve quantitation accuracy, and decrease run-to-run variability in proteomics studies with many biological and/or technical replicates.

Mass difference and isobaric labeling represent the primary tagging methods integrated into MS workflows, although some hybrid methods exist [16,17]. Mass difference labels incorporate discrete mass shifts metabolically [10] or chemically [18–20] onto proteins and peptides through the strategic use of heavy stable isotopes. They are lauded for providing accurate multiplexed quantification, but they are also criticized for increasing mass spectral complexity and reducing proteome coverage when compared to isobaric labels [21]. The advent of high-resolution mass difference labels solved this problem by incorporating subtle mass shifts onto peptides that can be elucidated with high-resolution mass analyzers [22–25], but high-resolution instruments are inaccessible to many labs.

Multiplexed quantification by isobaric labeling avoids the issues inherent in mass difference tagging by covalently bonding isotopic labels of identical mass to the N-terminus and lysine side chains of peptides in different samples. After combining samples, tandem mass spectrometry (MS^2^) methods fragment labeled peptides into both identifiable backbone product ions and discrete reporter ions. Reporter ion intensities are then compared to quantify the relative concentrations of differentially-labeled peptides. The sophisticated design of an isobaric label set is achieved by placing heavy isotopes onto each reagent’s reporter region and balancing the mass increase across labels by removing heavy isotopes from another region of the label. Isobaric labeling workflows have been successfully utilized to discover candidate biomarkers in multiple studies [4,15,26–28].

The power of isobaric labeling in quantitative proteomics is accompanied by a hefty financial burden. The two commercial products available, tandem mass tags (TMT) [29] and isobaric tags for relative and absolute quantitation (iTRAQ) [30], cost from $275 to $900 for each labeling experiment containing ~100 μg of protein digest per channel. High prices appear to be primarily due to production costs and vary depending on the multiplexing capacity of the reagent purchased. Our lab previously developed a novel, cost-effective 4-plex isobaric label, dimethylated leucine (DiLeu), and found its performance to be comparable to commercial reagents [31]. The DiLeu reagent can be synthesized in one to two steps at a yield of ~85%, and the material cost of labeling experiments is less than $5 total to label 100 μg of protein digest per channel. Later efforts utilized this label to increase the fragmentation efficiency of crab neuropeptides [32] and demonstrate an ion mobility technique to reduce co-isolation and co-fragmentation of isobarically-labeled *Escherichia coli* peptides [33]. However, the 4-plex DiLeu reagent has yet to be used in a study comparing protein abundances in disease and control samples. This study fills in that gap by utilizing DiLeu to quantify proteins from the urine of human males suffering from lower urinary tract symptoms (LUTS).

Lower urinary tract symptoms (LUTS) frequently afflict middle-aged and elderly men, negatively impacting their health and emotional state [34]. The financial burden of treating LUTS is approximately $3.9 billion per year, and costs are predicted to increase as the average age of the United States population climbs [35,36]. The totality of symptoms can be categorized into the obstructive and irritative [37]. Obstructive symptoms include hesitancy, straining, weak flow, prolonged voiding, partial or complete urinary retention, and overflow incontinence. Frequency, urgency, nocturia, painful urination, and small voided volumes comprise irritative symptoms [38,39]. Lower urinary tract symptoms have historically been linked to enlargement of the prostate, known as benign prostatic hyperplasia (BPH).

Histological BPH exists in 10% of men in their 30s, 20% of men in their 40s, 50% to 60% of men in their 60s, and 80% to 90% of men in their 70s and 80s [37]. Fibroblasts/myofibroblasts and epithelial glandular elements proliferate near the urethra at the transition zone of the prostate, resulting in its enlargement [40–45]. The definition of BPH has been expanded to include histological BPH, macroscopic glandular enlargement, and BPH-related symptoms and complications [46]. While this categorization is conveniently simple, it does not accurately represent the various contributors to LUTS [47,48]. For instance, not all men with histological BPH will experience LUTS, and some patients with LUTS do not have histological BPH [37]. Recent findings suggest overactive bladder (OAB) syndrome contributes to LUTS through detrusor overactivity and reduced detrusor contractility [39]. Abnormal muscle tone in the prostate and bladder neck could increase outlet resistance in the absence of prostate enlargement [49]. Prostatic inflammation has been strongly associated with LUTS through histological examination of human prostates [50–54] and mouse models [55–57]. Fibrosis also appears to play a key role in decreasing prostatic compliance and contributing to bladder outlet obstruction [55–61]. Other studies have highlighted the role that sex steroid hormones play in the development of BPH and/or LUTS [62], even modeling the hormonal milieu of aging males using dogs [63–66], rats [67,68], and mice [69,70]. The multiple and complex factors contributing to LUTS make it a relevant disease to characterize the ability of DiLeu isobaric labels to study changes in the urinary proteome.

## Experimental Section

### Chemicals and Reagents

Synthesis of DiLeu labels required purchasing the following isotopic reagents from ISOTEC Inc (Miamisburg, OH): leucines (ʟ-leucine and ʟ-leucine-1-^13^C, ^15^N), heavy formaldehyde (CD_2_O, 20% *w*/*w*), sodium cyanoborodeuteride (NaBD_3_CN), ^18^O water (H_2_^18^O), and deuterium water (D_2_O). Formaldehyde (CH_2_O, 37% *w*/*w*), sodium cyanoborohydride (NaBH_3_CN), trifluoroacetic acid (TFA), iodoacetamide (IAA), hydrogen chloride gas (HCl), tris hydrochloride, reagent-grade formic acid (FA), *N*,*N*-dimethylformamide (DMF), triethylammonium bicarbonate (TEAB), 4-(4, 6-dimethyoxy-1, 3, 5-triazin-2-yl)-4-methylmorpholinium tetrafluoroborate (DMTMM), sodium azide (NaN_3_) and *N*-methylmorpholine (NMM), and dithiothreitol (DTT) were purchased from Sigma-Aldrich (St. Louis, MO). Sequencing-grade trypsin was bought from Promega (Madison, WI). Urea, ACS grade methanol (MeOH), dichloromethane (DCM, CH_2_Cl_2_), acetonitrile (ACN, C_2_H_3_N), Optima LC-MS grade ACN, water, and FA were acquired from Fisher Scientific (Pittsburgh, PA). Hydroxylamine (H_3_NO, 50%) was purchased from Alfa Aesar (Ward Hill, MA).

### Synthesis of N,N-Dimethylated Leucine (DiLeu)

Synthesis of DiLeu reagents was previously described in detail [31]. Leucine was dimethylated by suspending either ʟ-leucine or ʟ-leucine-1-^13^C (99%), ^15^N (98%) in H_2_O or D_2_O (99.9% D) with a 2.5 molar excess of NaBH_3_CN or NaBD_3_CN (96% D). The reaction vessel was partially submerged in an ice-water bath. Light (CH_2_O) or heavy (CD_2_O) formaldehyde (98% D) was added at a 2.5 molar excess, the reaction vessel was sealed, and the mixture was stirred for 30 min. Amine dimethylation was observed with a ninhydrin stain on a thin-layer chromatography (TLC) plate. Dimethyl leucines were purified using flash column chromatography (DCM/MeOH) and dried.

### ^18^O Exchange

Labels corresponding to the 115 and 116 channels require ^18^O-exchange prior to formaldehyde dimethylation. ʟ-leucine or ʟ-leucine-1-^13^C, ^15^N was dissolved in 1N HCl H_2_^18^O solution (97% ^18^O, pH 1) and stirred at 65°C for 4 h. Acid was removed from the product using StratoSpheres PL-HCO_3_ MP resin (Agilent Technologies).

### DiLeu Activation to Triazine Ester Form

Two milligrams of each DiLeu label were dissolved in 50 μL of anhydrous DMF and combined with a 0.7x limiting molar ratio of DMTMM and NMM. It is crucial that no excess activating reagent exists after activation to obtain optimal labeling results. Activation occurred at room temperature by vortexing the reaction for 1 h. Labeling peptides immediately after DiLeu activation is essential for the best results.

### Human Patient Recruitment and Urine Collection

The urine sample collection at the University of Wisconsin Hospital has been approved by the UW-Madison Institutional Review Board (IRB). The IRB approval was obtained to specifically support this study with the ultimate goal to discover urinary biomarkers of LUTS in men. All participants provided their written informed consent to participate in this study. This consent procedure was approved by the Ethics Committee/IRBs. Midstream urine samples were collected from 25 consenting patients and 15 controls recruited by physicians and nurses in the Urology clinic at the University of Wisconsin Hospital according to the IRB Protocol. Patient inclusion and exclusion parameters are shown in Figure A in S1 File. Patients were men between the ages of 30–85 with significant LUTS under the treatment of alpha blocker therapy. Controls were male urology clinic patients without significant LUTS who were undergoing follow-up visits for renal cell carcinoma, renal cystic disease, kidney stones, erectile dysfunction, low-grade prostate cancer, hydrocele, or were on a watchful waiting protocol. After collection, urine samples were de-identified, spiked with sodium azide, and stored at -80°C.

### Urine Sample Preparation

Two milliliter aliquots of urine were concentrated to 0.5 mL and centrifuged at 10000g for 10 min. Amicon ultra-0.5 mL centrifugal filters (3 kDa) purchased from EMD Millipore (Billerica, MA) were used to separate urinary proteins from small molecules according to the manufacturer’s protocol. Proteins captured on the filter were washed twice at 14000g for 30 min with water to remove possible interferences. Total protein concentration was measured from each sample via a Thermo Scientific BCA Protein Assay Kit (Rockford, IL) at an absorbance of 570 nm using a Tecan Ultra 384 microplate reader (Männedorf, Switzerland). Urinary protein samples were each normalized to 200 μg and further concentrated.

### Urinary Protein Reduction, Alkylation, and Digestion

Urinary proteins were dissolved in 8 M urea/50 mM Tris HCl (pH 8). Disulfide bonds were reduced with 5 mM DTT for 1 h at room temperature. Free thiol groups were alkylated in the dark using 15 mM IAA at room temperature for 15 min before quenching with 5 mM DTT. Protein samples were then diluted with 50 mM Tris HCl until reaching a urea concentration of < 1 M before adding trypsin in a 50:1 protein:enzyme ratio. Proteins were digested at a temperature of 37°C for 16 h. Digests were quenched by lowering the pH to <3 with 10% TFA. Peptides were desalted with SepPak C_18_ solid-phase extraction (SPE) cartridges (Waters, Milford, MA) according to the manufacturer’s protocol, concentrated, and reconstituted in 0.5 M TEAB before labeling.

### Urinary Peptide Labeling with 4-plex DiLeu

Human urine peptides were labeled with a 5x *w*/*w* excess of DiLeu. Channel randomization ensured that patient and control samples were labeled with different reagent channels for each LC-MS/MS run. Anhydrous DMF was added to the reaction mixture so that the organic:aqueous ratio reached 70%. The labeling reaction was shaken for 2 h and quenched with 0.25% *v*/*v* hydroxylamine. Labeled urine samples were then dried.

### Strong Cation Exchange Fractionation

Labeled urine peptides were dissolved in 10 mM KH_2_PO_4_, 20% ACN (*v*/*v*), pH 3. Peptides were separated from DiLeu reaction byproducts and fractionated by charge with strong cation exchange chromatography (SCX) using a polySULFOETHYL A 200 mm x 2.1 mm, 5 μm, 300 Å column (PolyLC, Columbia, MD) on a Waters Alliance e2695 HPLC (Milford, MA). Buffer A was composed of 10 mM KH_2_PO_4_, 20% ACN (*v*/*v*), pH 3, and buffer B consisted of 10 mM KH_2_PO_4_, 500 mM KCl, 20% ACN (*v*/*v*), pH 3. Peptides were loaded onto the column, and B increased from 0–33% over 75 min and then to 100% over the next 25 min at a flow rate of 0.2 mL/min. Fractions were collected every 1.5 min and reduced into four vials determined by UV-Vis at 215 nm. All samples were dried and re-dissolved in 0.1% TFA before being desalted with C_18_ OMIX pipette tips (Agilent Technologies, Santa Clara, CA).

### LC-MS^2^ Acquisition

Labeled urine peptide samples were dissolved in 0.1% FA and separated with a Waters nanoAcquity UPLC before entering a Thermo Q-Exactive Orbitrap mass spectrometer (San Jose, CA). Each sample was injected twice. Mobile phase A consisted of water with 0.1% FA, and mobile phase B was composed of ACN with 0.1% FA. Samples were loaded onto a fabricated column with an integrated emitter. The 75 μm ID column was filled to a length of 15 cm using Ethylene Bridged Hybrid C_18_ packing material (1.7 μm, 130 Å, Waters). Peptides were trapped onto the column in 100% A and separated using a solvent gradient of 0–10% B over 0.5 min and then 10–30% B over 70 min at a flow rate of 350 nL/min. Data-dependent acquisition (DDA) parameters recorded MS scans in profile mode from *m*/*z* 380–1500 at a resolution of 35K. Automatic gain control (AGC) targets of 1 x 10^6^ and maximum injection times (IT) of 100 ms were selected. The 15 most intense precursor ions were selected for MS^2^ higher-energy collisional dissociation (HCD) fragmentation with an isolation width of 2.0 *m*/*z* and placed on an exclusion list for 40 s. Tandem mass spectra were acquired at a resolution of 17.5K in profile mode with an AGC target of 1 x 10^5^, a maximum IT of 150 ms, a normalized collision energy (NCE) of 27, and a fixed lower mass at *m*/*z* 110.

### Data Analysis

Labeled urinary tryptic peptides were identified using the Proteome Discoverer software suite (1.4.0288, Thermo Scientific). Raw files were searched against a *Homo sapiens* reference database obtained from UniProt using the SEQUEST HT algorithm. Tryptic peptides with at most two missed cleavages were matched using precursor and fragment mass tolerances of 50 ppm and 0.02 Da, respectively. Three static modifications, cysteine carbamidomethylation (+57.0215 Da), N-terminus DiLeu labeling (+145.1280 Da), and lysine residue DiLeu labeling (+145.1280 Da), were chosen. The only variable modification chosen was methionine oxidation (+15.9949 Da). Peptide spectral matches (PSMs) were validated from q-values set to a false discovery rate (FDR) of 1% using Percolator [71]. Reporter ion intensities from DiLeu-labeled peptides were collected from raw files with Proteome Discoverer at a reporter ion integration tolerance of 20 ppm for the most confident centroid. Peptide spectral matches with all four channels present were considered valid for quantification. Proteins characterized by one unique peptide were considered valid matches if identified in at least eight samples and at least three PSMs from unique peptides were available for quantification (see S3 File for MS^2^ spectra). Reporter intensity averages were exported to Excel, where label impurities were accounted for by using correction factors determined with a previously described method [72] and imported from PTC Mathcad 14 (Needham, MA). Generated equations are shown in Figure B in S1 File. Quantification values for each protein were mean normalized to enable comparison across biological replicates, and these values were normalized again using the mean of each channel to account for minor differences of total protein content in each channel. Normalized signals corresponding to LUTS patients for each protein were averaged in each run and compared against the average control signals from each run. Quantified proteins were subjected to a two-sample unequal variance Student’s t-Test with a two-tailed distribution. Proteins with abundance changes >20% and p-values <0.05 were placed on a list for further study.

### GO-term Enrichment Analysis

Gene ontology (GO) enrichment analysis of the 50 differentiated proteins was performed using the Database for Annotation, Visualization, and Integrated Discovery (DAVID) v6.7 (http://david.abcc.ncifcrf.gov/) [73]. Gene groups with enrichment scores ≥1.3 were explored. Protein set enrichment analysis (PSEA-Quant) was also used to analyze the protein quantification dataset [74]. Abundance ratios were input into the online PSEA-Quant interface. The Gene Ontology annotation database was selected, protein abundance dependence was assumed, a coefficient of variation tolerance factor of 0.5 was input, and protein annotation bias was also assumed.

## Results and Discussion

### Characteristics of 4-plex DiLeu

**Fig 1:**
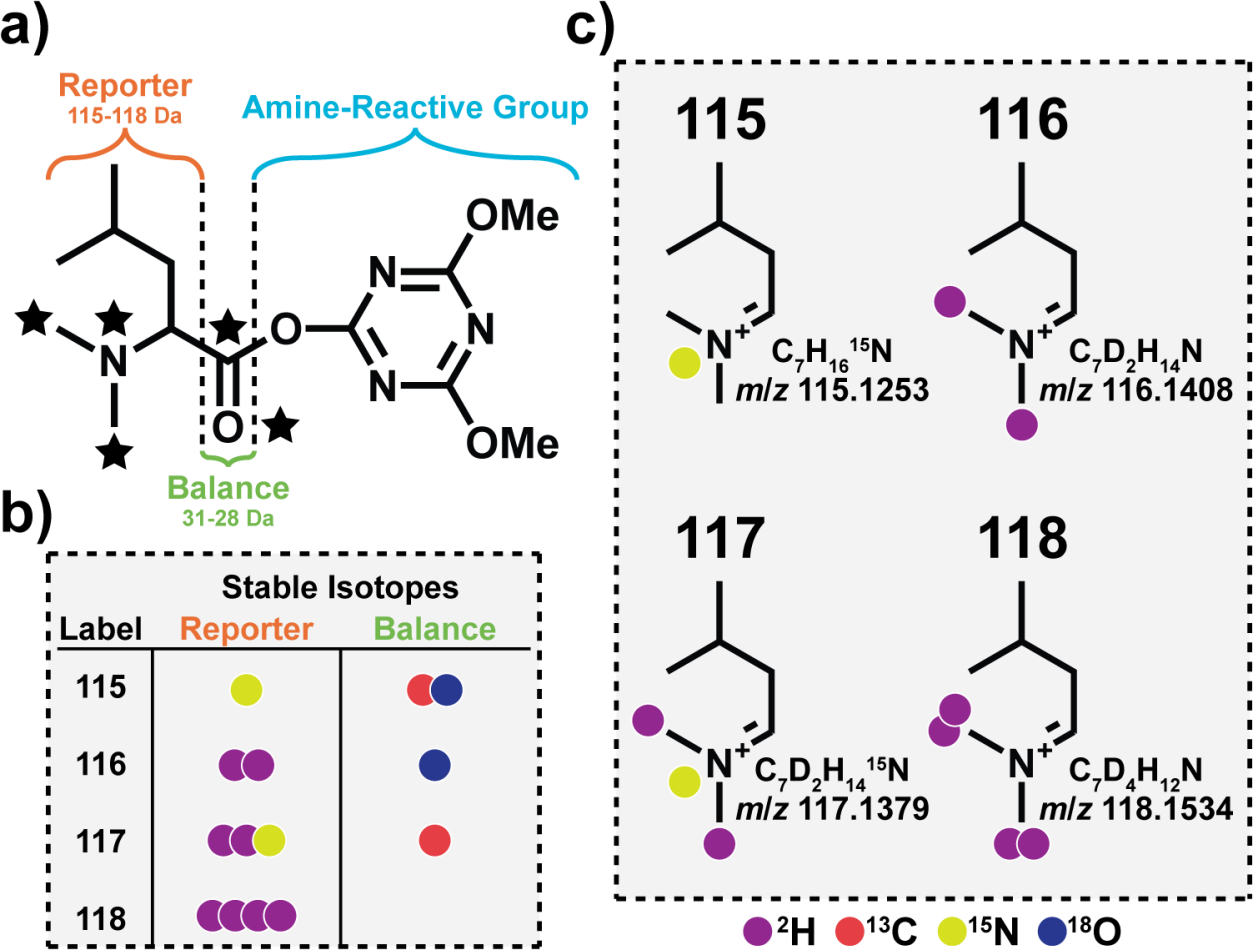
N, N- Dimethylated Leucine (DiLeu) as multiplexed isobaric tags for relative quantitation. a) DiLeu Structures, b) Isotopic Incorporations, and c) Reporter Ions.

**Fig 2:**
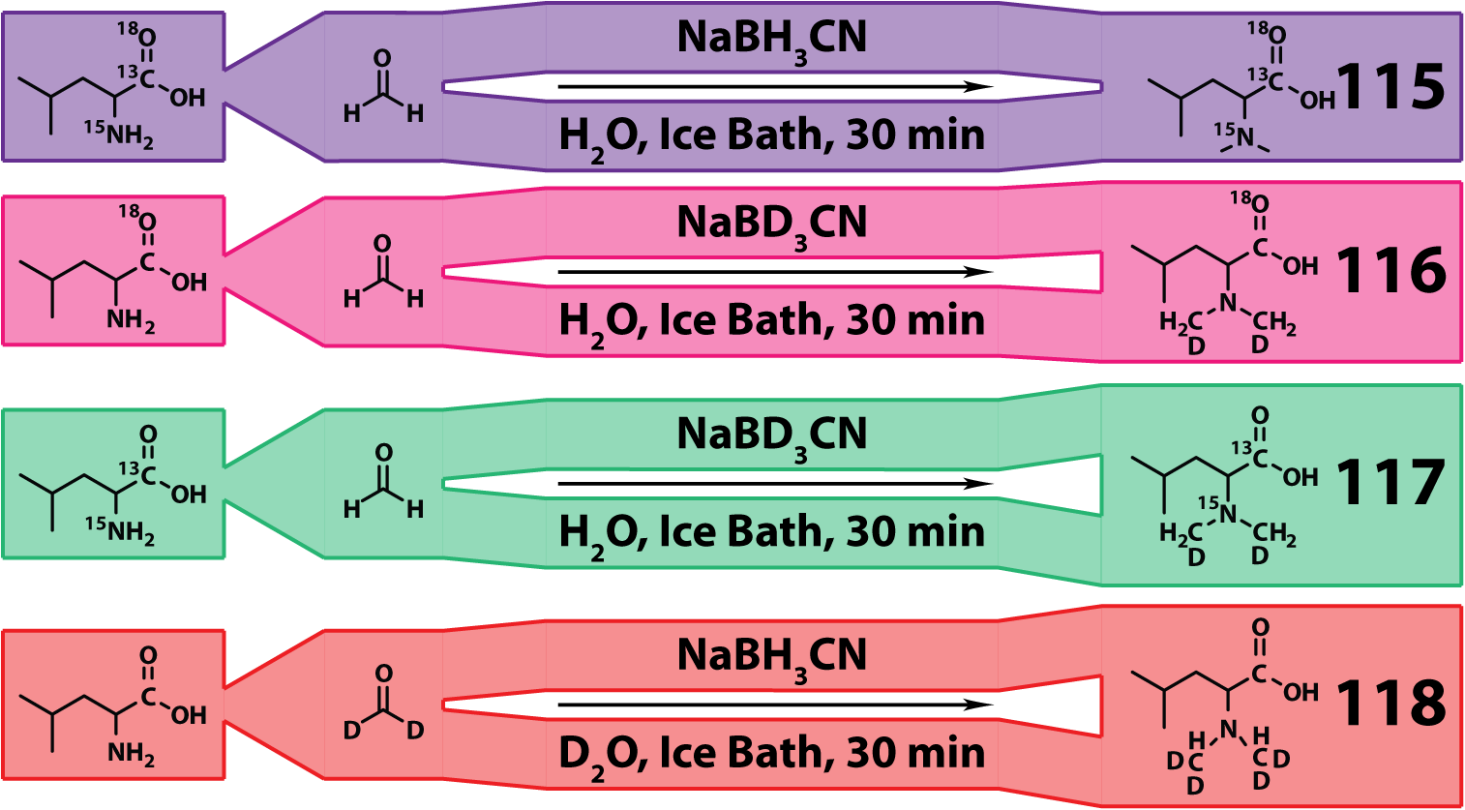
Synthesis of 4-plex DiLeu. DiLeu is synthesized in a simple 1–2 step process.

DiLeu reagent and reporter ion structures are shown in Fig 1. Heavy isotopes, ^2^H, ^13^C, ^15^N, ^18^O are strategically placed onto the DiLeu structure to create an isobaric 4-plex reagent set. The simple reactions utilized to synthesize DiLeu are displayed in Fig 2. Covalent bonding of DiLeu to peptide N-termini and lysine residues adds a relatively modest mass of ~145.1 Da for each site labeled. Attached DiLeu tags fragment into dimethyl immonium ions upon HCD or collisional induced dissociation (CID) with *m*/*z* values of 115.1523, 116.1408, 117.1379, or 118.1534. A carbonyl balance group leverages the increasing reporter region mass by replacing ^13^C and ^18^O isotopes with ^12^C and ^16^O. Triazine ester is the preferred amine-reactive group over N-hydroxysuccinimide (NHS) ester because of its high reactivity and efficiency of DiLeu activation. The original 4-plex DiLeu report showed that this reagent set is cost effective and simple to synthesize. Further observations proved that DiLeu efficiently labels peptides (~99.9% conversion) and reporter ion intensities accurately reflect their relative abundances. The previous study also found that deuterium retention time shifts of labeled peptides were negligible and efficient fragmentation occurred at slightly increased CID energies [31,75,76].

### Performance of 4-plex DiLeu in Quantitative Proteomics using LUTS Urinary Samples

**Fig 3:**
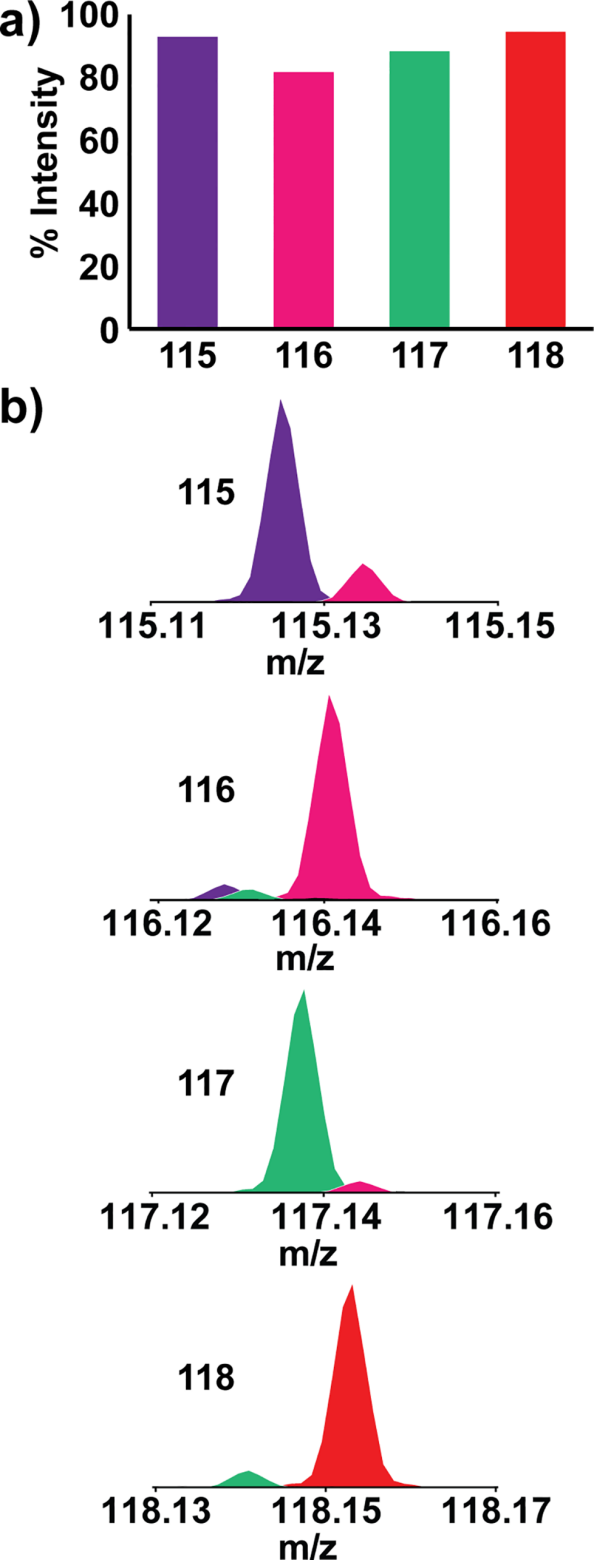
Isotopic impurities in 4-plex DiLeu reagents. a) Isotopic impurities in 4-plex DiLeu reagents cause reporter ion signals at 1:1:1:1 ratios to be slightly lower and differ from one another (0.92:0.81:0.88:0.94) in intensity. Correction factors are applied to account for these differences. b) An MS^2^ resolution of 17.5K allows elucidation of reporter ion impurities from signals while maintaining a rapid MS^2^ acquisition speed.

Factors measuring 4-plex DiLeu labeling performance were evaluated previously [31], but we addressed some outstanding characteristics related to protein quantification in complex biological fluids here. DiLeu reagents are synthesized with commercially-available high-purity reagents, but the slight isotopic impurities in these chemicals, reported above, influence reporter ion signal intensity and must be corrected for. The experimental purity of each label channel is shown by Fig 3. When labeled peptides are mixed together at a theoretical ratio of 1:1:1:1, reporter ion abundances correspond in a ratio of ~0.92:0.81:0.88:0.94 because of these impurities. The fractions absent from each reporter signal can be observed ±1 *m*/*z* from each reporter ion and influence reporter ion quantification at low MS^2^ resolutions. Fig 3 reveals that an MS^2^ resolution of 17.5K sufficiently resolved DiLeu quantification channels from potential interferences. Thus, at a resolution of 17.5K, DiLeu reporter ion signal correction factors do not need to account for interference. Instead, corrections add impurity intensities to the raw reporter ion signals. The final equations for corrections are shown in Figure B in S1 File.

**Fig 4:**
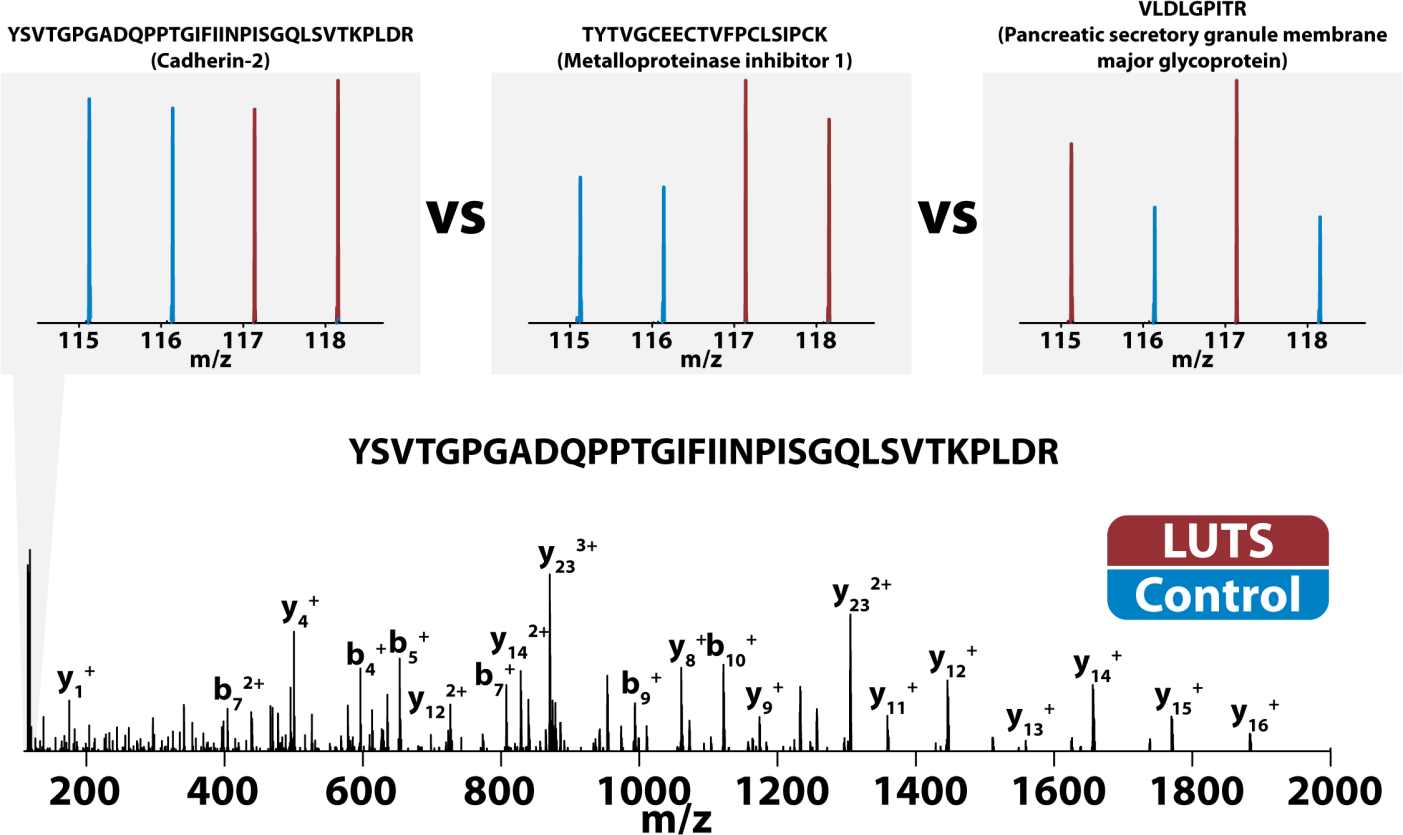
Fragmentation of DiLeu-labeled urinary peptides. Fragmentation of DiLeu-labeled urinary peptides yielded rich b- and y- product ion MS^2^ spectra. Reporter ion intensities were compared across PSMs to determine if proteins were up- or down-regulated.

DiLeu-labeled peptides were fragmented at higher-than-typical CID energies in the past [31]. We have found that using a slightly lower normalized collision energy (NCE) than average for HCD fragmentation on Orbitrap instruments yields rich product ion spectra and intense reporter ion signals. Tagged urinary peptides fragmented well at an NCE of 27, and Fig 4 shows an MS^2^ spectrum of a DiLeu-labeled peptide. This spectrum shows a plethora of b- and y- product ions matched to a peptide sequence by the SEQUEST algorithm. The mean cross-correlation (XCorr) value calculated from 66090 MS^2^ spectral matches was 3.01(±1.31). An XCorr score is dependent on the quality of the MS^2^ spectrum and its similarity to the predicted spectrum [77], and a score of three is generally a high-quality match. Examples of reporter ion quantification are also given in Fig 4. Reporter ion signal intensities belonging to the peptide YSVTGPGADQPPTGIFIINPISGQLSVTKPLDR are nearly equal and suggest that the corresponding protein, cadherin-2, is not up- or down-regulated in LUTS patients. The peptides TYTVGCEECTVFPCLSIPCK and VLDLGPITR are both up-regulated according to the DiLeu reporter ion signals shown by Fig 4. These peptides were mapped to metalloproteinase inhibitor 1 and pancreatic secretory granule membrane major glycoprotein, respectively.

Each 4-plex labeling experiment contained peptides from four different human patients. After SCX fractionation, peptides were combined into four vials, and each of these vials was injected twice for LC-MS^2^ analysis. Proteome Discoverer was used to combine the 80 LC-MS^2^ runs, resulting in the identification and quantification of 836 proteins from 4426 peptides matched at an FDR ≤ 1%. Proteins matched by only one unique peptide had to be detected in eight patients with at least three PSMs used for quantification. Figure C in S1 File plots proteins by the sum of reporter ion intensities from all LC-MS^2^ runs versus their average log_2_ transformed LUTS:control protein ratios. The majority of proteins (730), regardless of reporter ion sum, have a log_2_ ratio translating to less than a 20% change. This result was expected because it is probable that most urinary proteins are not involved in LUTS, and many protein quantification studies find that a high percentage of proteins are not up- or down-regulated. Some DiLeu-labeled proteins have smaller reporter ion sums because they were quantified with lower reporter ion abundances and/or fewer PSMs. These cases were still normally distributed around log_2_ ratios of 0, and proteins quantified at intermediate abundance sums between 10^7^ and 10^8^ accounted for the majority of up- or down-regulated proteins. Furthermore, only 7% of the proteins identified by one unique peptide passed the criteria (>20% change, p-value < 0.05) for up- or down-regulation, suggesting that proteins quantified with fewer unique peptides, PSMs, and/or less intense reporter ions still produce valid quantitative data.

Figure D in S1 File shows that urinary proteins quantified with 4-plex DiLeu follow a Gaussian distribution. Proteins that passed the expression cutoff (20% abundance change, p-value < 0.05) were highlighted in pink. P-values were determined by a two-sample unequal variance Student’s t-Test with a two-tailed distribution. Forty proteins were found to be up-regulated, and 10 proteins were down-regulated. The variability in human samples increased the number of proteins showing >20% change, but filtering by p-values ensured that only statistically relevant proteins were examined. Less dramatic changes in protein abundance were examined because of the known ratio compression that occurs from co-isolation of isobarically-labeled peptides [21,78]. Methods to mitigate ratio compression include ion mobility, MS^3^, and gas-phase purification, but none of them have reached widespread acceptance due to the drawbacks they introduce or the specialized instrumentation they require [33,79–83]. Up- and down-regulated proteins were further examined through GO-term enrichment analysis.

### Evaluation of Quantified Proteins

**Fig 5:**
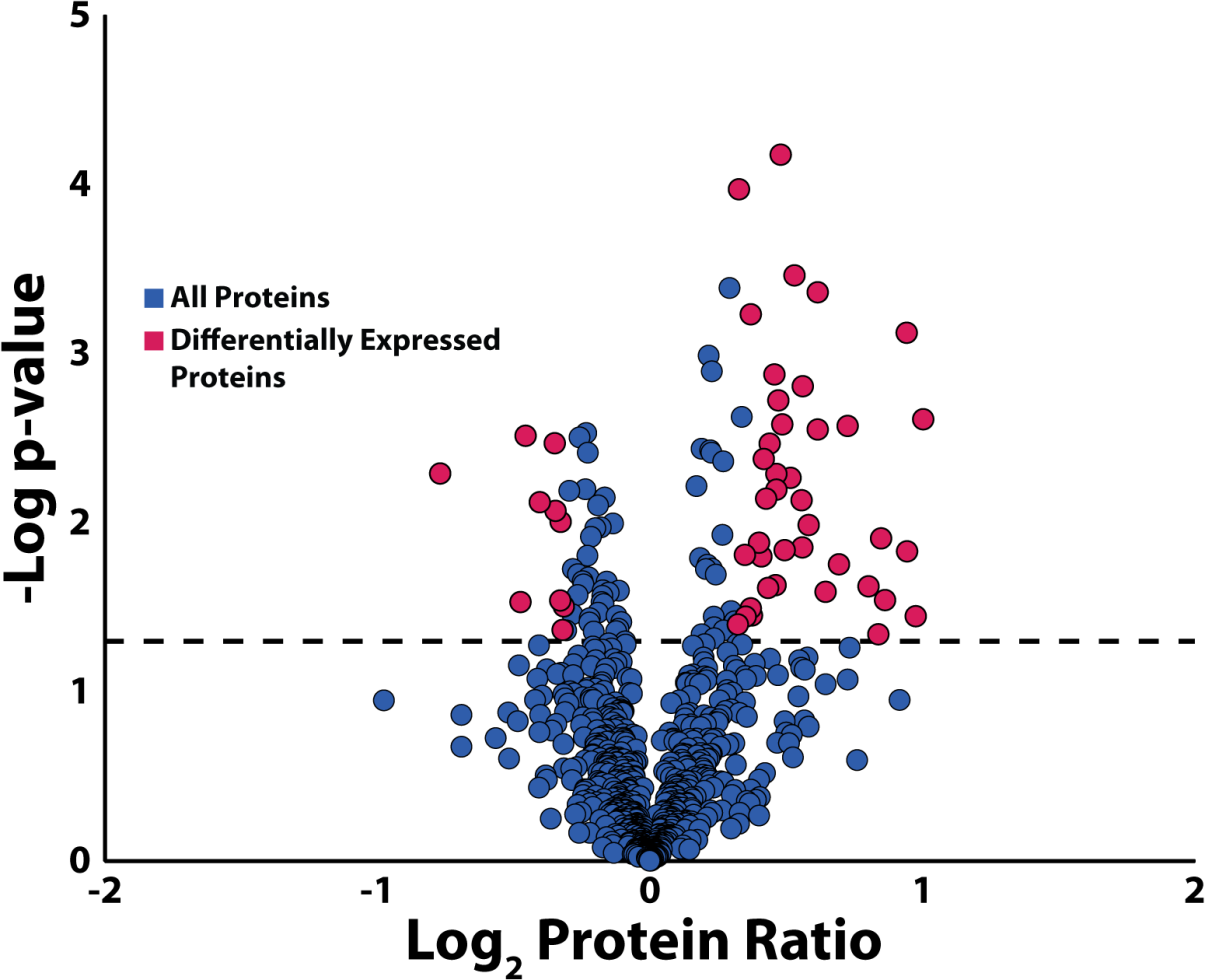
Volcano plot of quantified urinary proteins. The volcano plot of quantified urinary proteins reveals that 50 such proteins showed expression in LUTS patients compared to control samples. Proteins with fold-changes of 20% and p-values < 0.05 are shown in pink. Each protein was further characterized through literature searches and GO-term enrichment analysis.

The 836 proteins quantified included 50 that were further assessed using GO-term enrichment analysis. Patients with LUTS were already undergoing alpha-blocker treatment at the time of urine collection while controls were not. The possibility exists that some proteins were differentially expressed due to alpha-blocker therapy. S1 Table contains pertinent information regarding each protein like UniProt accession number, gene symbol, p-value, average mean normalized signals, ratio, coefficient of variation (CV), and number of unique peptides used to quantify each protein. The–log_10_ p-value of each protein was plotted against log_2_ protein ratio to construct a volcano plot measuring the statistical significance and magnitude of fold change for each protein (Fig 5). Proteins colored in pink are statistically significant (p-value < 0.05) and have fold changes greater than 20%. These proteins were investigated further through literature searches.

Nine of the differentially expressed proteins have been extensively implicated in fibrosis or fibrosis disorders. Amiloride-sensitive sodium channel subunit gamma (SCNN1G) has been reported to be a relevant factor in fibrosis through disruption of the amiloride-sensitive sodium channel ENaC [84,85]. Cytokeratin 14 (KRT14) has been suggested as a diagnostic marker for oral submucous fibrosis (OSF) in the oropharynx [86] and could also play a role in prostate fibrosis. Fibroblast proliferation and extracellular matrix deposition by the endothelin system, which endothelin-3 (EDN3) is part of, contribute to the progression of fibrosis [87,88]. An increase in metalloproteinase inhibitor 1 (TIMP1) while levels of 72 kDa type IV collagenase (MMP2) remain low has been correlated to the development of fibrosis in the liver, and a further increase of MMP2 predicted the onset of liver cirrhosis [89]. This finding suggested that the interplay between metalloproteinase and inhibitor may be more complex in fibrotic disorders than their nomenclature suggests. Investigation of renal fibrosis by oxidative stress pathways resulted in the identification of Peroxiredoxin-2 (PRDX2) and Protein DJ-1 (PARK7) as potential biomarkers [90,91], corroborating our data. Cystic fibrosis transmembrane conductance regulator knockout mice have been utilized to reveal that the gene encoding deleted in malignant brain tumors 1 protein (DMBT1) plays a role in fibrosis [92]. One interesting finding in our study is the decrease in Collagen alpha-1(XIV) (COL14A1) for LUTS patients. Collagens typically increase during the onset of fibrotic disorders, but COL14A1 helps regulate fibril diameter [93]. Studies have suggested that such collagens may actually decrease during certain stages of fibrosis, allowing fibrils to fuse to larger-diameter structures and contribute to fibrosis progression [93,94].

Numerous other up- or down-regulated proteins were found to be biologically interesting. Studies suggesting either primary or periphery roles in fibrosis were also found for the following proteins: cytokeratin 16 (KRT16) [95], tumor necrosis factor receptor superfamily member 12A [84], anosmin-1 (KAL1) [96], protein S100-A4 (S100A4) [97], trefoil factor 3 (TFF3) [98], coagulation factor XII (F12) [99], fatty acid-binding protein (FABP1) [100], prostaglandin reductase 1 (PTGR1) [101], and alpha-2-antiplasmin (SERPINF2) [102]. A considerable number of other proteins were involved in disorders related to urination but not necessarily to inflammation or fibrosis. These proteins include midkine (MDK) [103], transthyretin (TTR) [104], plastin-3 (PLS3) [105], heat shock protein beta-1 (HSPB1) [106], sodium/potassium-transporting ATPase subunit gamma (FXYD2) [107], prostatic acid phosphatase (ACPP) [108], complement component C6 (C6) [109], protein phosphatase 1 regulatory subunit 37 (PPP1R37) [110], retinal dehydrogenase 1 (ALDH1A1) [111], and cystatin-A (CSTA) [112]. It may seem odd that semenogelin-1 and 2 (SEMG1 and SEMG2) were found to be up-regulated, but previous studies linked sexual and ejaculatory dysfunction to LUTS [113,114].

### GO-Term Enrichment Analysis

Computational approaches to analyze quantitative proteomics results help cluster protein lists into enriched sets according to their functional annotations. Metabolic and signaling pathways are rarely found to be enriched in urine [2]. However, GO-term enrichment of differentially expressed proteins determined from isobaric labels can help substantiate claims regarding protein function. Functional annotation clustering of up-regulated proteins using the DAVID Bioinformatics Database returned five annotation clusters with enrichment scores ≥ 1.3 [73]. As expected, most proteins were found to belong to the cellular component, extracellular region. The most significantly enriched biological processes were shown to be acute inflammatory response, response to wounding, and inflammatory response. Four genes, coagulation factor XII, complement component 6, peroxiredoxin 2, and alpha-2-antiplasmin, were involved in acute inflammatory and inflammatory response. Genes playing a role in response to wounding were coagulation factor XII, complement component 6, midkine, peroxiredoxin 2, alpha-2-antiplasmin, and amiloride-sensitive sodium channel subunit gamma. Functional annotation clustering of down-regulated urinary proteins once again found that these factors were relegated to the extracellular region, but no enriched biological processes were found.

**Fig 6:**
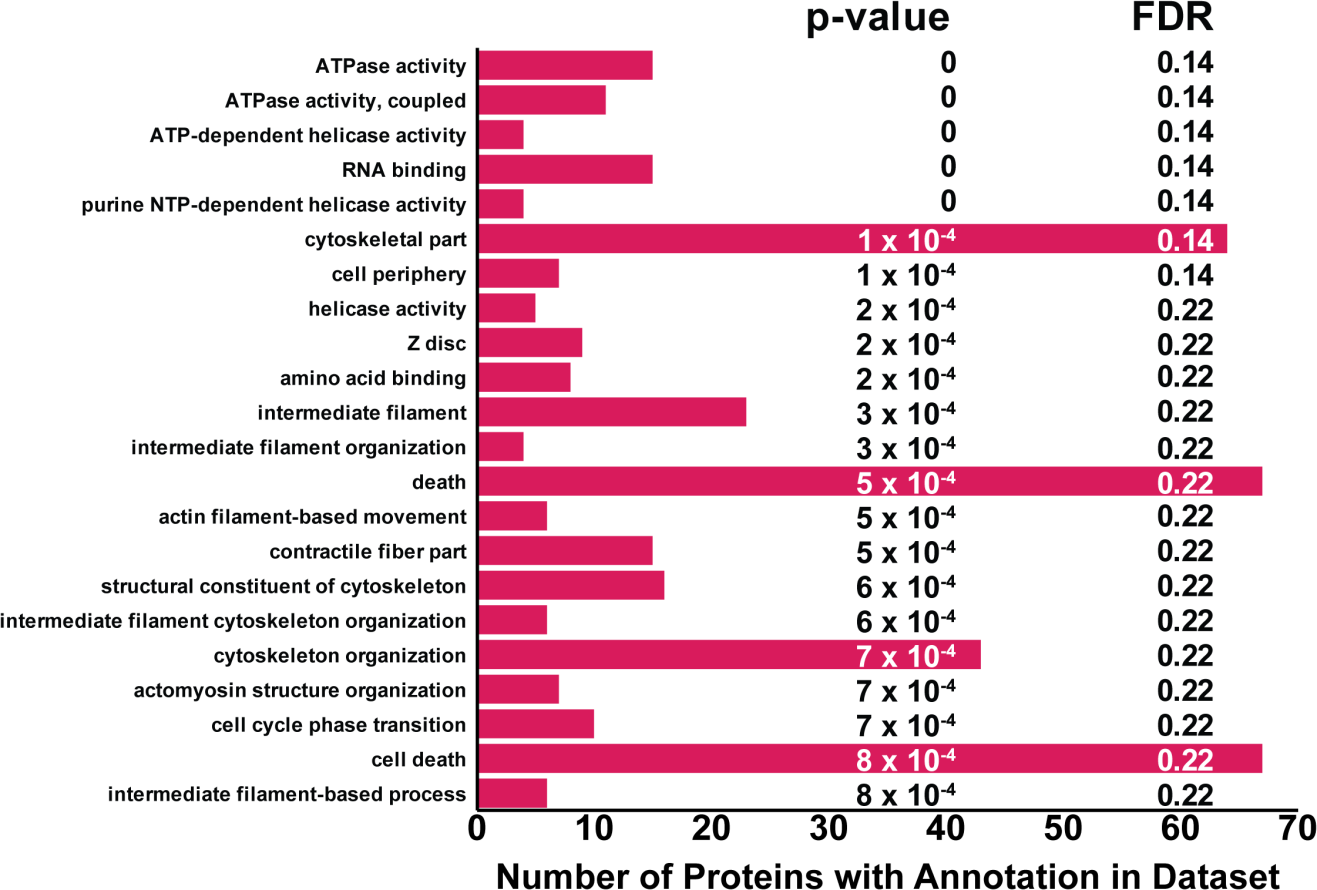
PSEA-Quant analysis of quantified protein set. PSEA-Quant analysis generated complementary GO processes when compared with DAVID. Protein numbers on the x-axis represent the complete set of proteins found in the dataset associated with a particular GO-term. Many processes and cellular component descriptions like cytoskeletal part, Z disc, intermediate filament organization, and cytoskeleton organization have been implicated in fibrosis. GO results suggested that fibrosis plays a key role in the onset and pathogenesis of LUTS.

Traditional GO analysis suffers from the following limitations: 1) expressed genes/proteins are considered to be independent of each other, 2) accurate protein abundance measurement is unaccounted for, and 3) arbitrary thresholds (p-value, fold-change) determine which proteins are submitted for analysis [74]. A new method, the protein set enrichment analysis tool (PSEA-Quant), statistically assesses the enrichment of proteins using protein quantification results from replicated experiments for a single or multiple conditions. This algorithm uses a permutation scheme to model protein abundance dependencies and annotation biases without requiring arbitrary thresholds for submitted features-of-interest. The entire protein dataset and quantitative ratios can be submitted to PSEA-Quant. The developers of PSEA-Quant showed that it yielded results complementary to classic GO-term enrichment methods and works well using label-free and label-based protein quantification methods [74]. Our goal was to use this new technique not as a replacement to traditional GO analysis, but as a complementary method. When we applied PSEA-Quant to our LUTS dataset, several GO-terms, many potentially related to prostate fibrosis, were enriched. **Fig 6** is a bar chart comparing the number of proteins per enriched GO-term. Terms like cytoskeletal proteins, filament proteins, cytoskeletal organization, filament organization, and actin filament-based movement may all be related to fibrosis as filament organization and rearrangement is thought to play a role in its development [115–117]. Determination of enriched protein sets using PSEA-Quant found biological processes in LUTS complementary to those from DAVID.

## Conclusions

We have developed a relative quantification strategy using our custom, noncommercial isobaric labeling reagents, DiLeu, capable of studying proteomic changes to assist in solving complex clinical problems. While the differentially expressed proteins are not meant to be interpreted as biomarker candidates without extensive confirmatory investigations, the quantitative strategy reported here indicates DiLeu’s potential in quantitative studies of biologically relevant systems. Furthermore, this research provides a guide for labs interested in synthesizing and applying their own noncommercial quantitative labels. In the future, our quantitative labeling workflow will quantify differentially expressed proteins found in this study using our custom label for absolute quantification, iDiLeu [75]. For the first time, a noncommercial isobaric label, DiLeu, has been used to quantify proteins from clinically relevant samples, providing an economical solution for future isobaric labeling studies that does not depend on the purchase of costly commercial labels.

## Supporting Information

S1 File Supplemental figures referenced within manuscript text. Inclusion and exclusion parameters used to recruit LUTS patients (**Figure A in S1 File**). a) Raw signal (S) is a product of the fraction of the pure reporter ion (x) and the actual reporter ion abundance (I). Equations are rearranged in b) to solve for I (**Figure B in S1 File**). Relative quantification of DiLeu-labeled urinary proteins in LUTS vs control patients showed that, as expected, most proteins are neither up- nor down-regulated. A total of 836 proteins, identified by at least three PSMs and one unique peptide in two runs, were quantified. Proteins quantified with a lower reporter ion sum due to fewer PSMs or lower reporter ion abundances are still distributed around ratios close to unity, meaning that their quantitative results are most likely valid (**Figure C in S1 File**). Ratios of DiLeu-labeled urinary proteins follow a Gaussian distribution around unity ratios. Proteins with abundance changes of ±20% were further filtered by p-values (< 0.05) and are shown in pink (**Figure D in S1 File)**.
(DOCX)

S2 File DiLeu quantification ratios for identified proteins (XLSX)

S3 File Tandem mass spectra of peptides for proteins identified with one unique peptide. (DOCX)

S1 Table Differentially expressed proteins determined by DiLeu labeling of LUTS urine. (DOCX)
