# Supplementary material for: Custom 4-Plex DiLeu Isobaric Labels Enable Relative Quantification of Urinary Proteins in Men with Lower Urinary Tract Symptoms (LUTS)
Source: PLoS One. 2015 Aug 12;10(8):e0135415. doi: 10.1371/journal.pone.0135415 (PMC4534462; doi:10.1371/journal.pone.0135415)
Supplement: S1 File — Inclusion and exclusion parameters used to recruit LUTS patients (Figure A in S1 File). a) Raw signal (S) is a product of the fraction of the pure reporter ion (x) and the actual reporter ion abundance (I). Equations are rearranged in b) to solve for I (Figure B in S1 File). Relative quantification of DiLeu-labeled urinary proteins in LUTS vs control patients showed that, as expected, most proteins are neither up- nor down-regulated. A total of 836 proteins, identified by at least three PSMs and one unique peptide in two runs, were quantified. Proteins quantified with a lower reporter ion sum due to fewer PSMs or lower reporter ion abundances are still distributed around ratios close to unity, meaning that their quantitative results are most likely valid (Figure C in S1 File). Ratios of DiLeu-labeled urinary proteins follow a Gaussian distribution around unity ratios. Proteins with abundance changes of ±20% were further filtered by p-values (< 0.05) and are shown in pink (Figure D in S1 File). (DOCX) [file pone.0135415.s001.docx]

**S1 File**


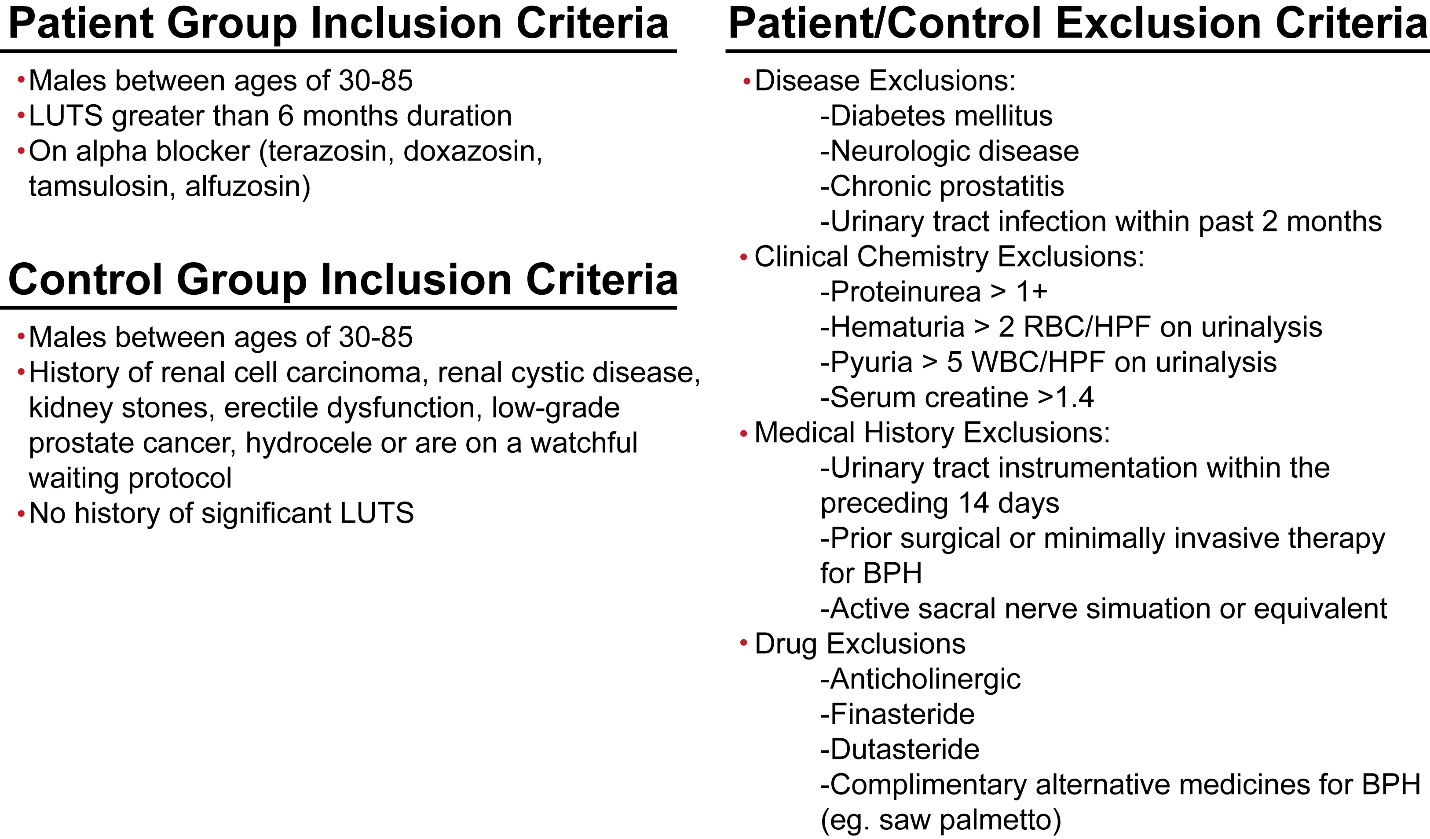


**Figure A.** **Inclusion and exclusion parameters used to recruit LUTS patients**


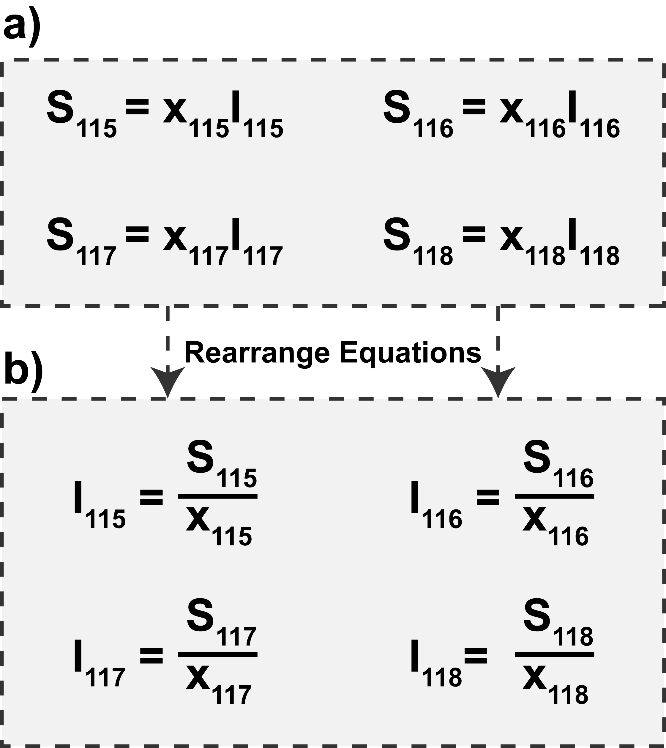


**Figure B.** a) Raw signal (S) is a product of the fraction of the pure reporter ion (x) and the actual reporter ion abundance (I). Equations are rearranged in b) to solve for I.


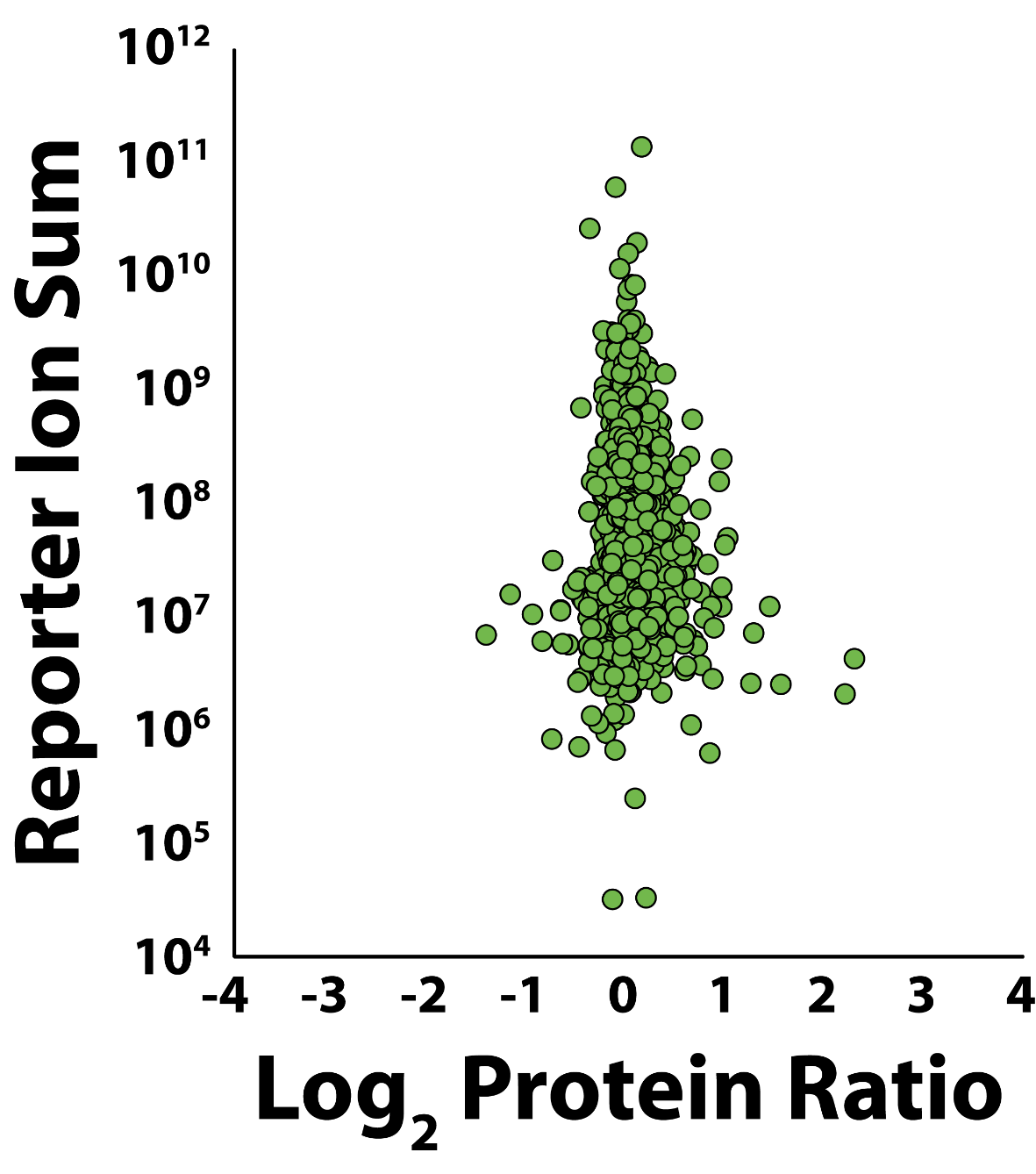


**Figure C.** Relative quantification of DiLeu-labeled urinary proteins in LUTS vs control patients showed that, as expected, most proteins are neither up- nor down-regulated. A total of 836 proteins, identified by at least three PSMs and one unique peptide in two runs, were quantified. Proteins quantified with a lower reporter ion sum due to fewer PSMs or lower reporter ion abundances are still distributed around ratios close to unity, meaning that their quantitative results are most likely valid.

**
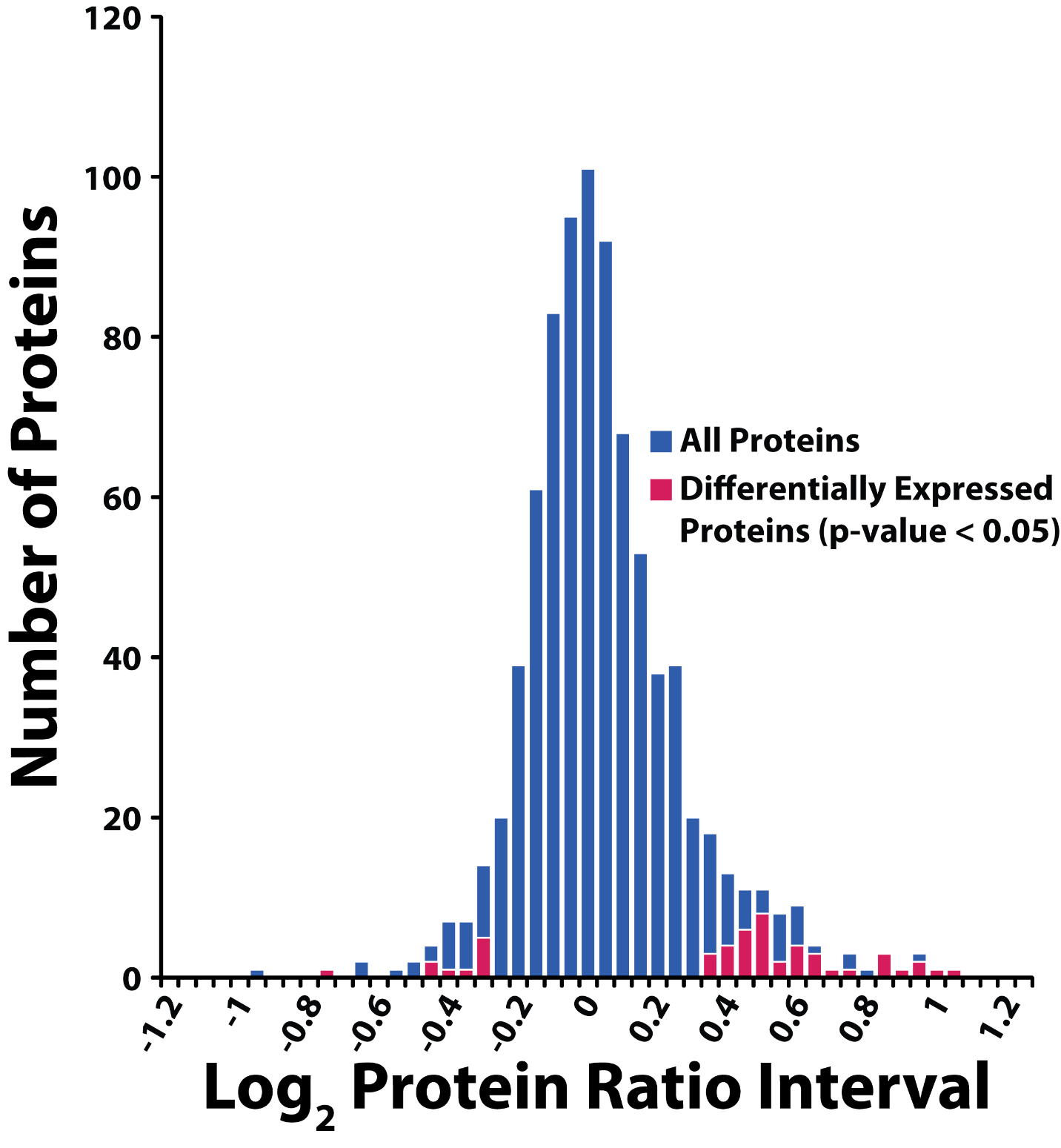
**

**Figure D.** Ratios of DiLeu-labeled urinary proteins follow a Gaussian distribution around unity ratios. Proteins with abundance changes of ±20% were further filtered by p-values (< 0.05) and are shown in pink.
