# Supplementary material for: Custom 4-Plex DiLeu Isobaric Labels Enable Relative Quantification of Urinary Proteins in Men with Lower Urinary Tract Symptoms (LUTS)
Source: PLoS One. 2015 Aug 12;10(8):e0135415. doi: 10.1371/journal.pone.0135415 (PMC4534462; doi:10.1371/journal.pone.0135415)
Supplement: S3 File — (DOCX) [file pone.0135415.s003.docx]

**S3 File. One unique peptide spectra.**

Ig kappa chain V-I region AU OS=Homo sapiens PE=1 SV=1 - [KV102_HUMAN]

vTITcQASQDISDYLNWYQQkPGk (+4.62 ppm)

Ig kappa chain V-II region Cum OS=Homo sapiens PE=1 SV=1 - [KV201_HUMAN]

eDIVMTQTPLSLPVTPGEPASIScR (+17.85 ppm)

Ig kappa chain V-II region TEW OS=Homo sapiens PE=1 SV=1 - [KV204_HUMAN]

dIVmTQSPLSLPVTPGEPASIScR (+7.37 ppm)

Ig kappa chain V-I region Rei OS=Homo sapiens PE=1 SV=1 - [KV115_HUMAN]

lLIYEASNLQAGVPSR (-8.07 ppm)

Ig kappa chain V-I region DEE OS=Homo sapiens PE=1 SV=1 - [KV105_HUMAN]

yLNWYQQkPGk (+7.44 ppm)

Ig kappa chain V-I region Ka OS=Homo sapiens PE=1 SV=1 - [KV111_HUMAN]

dIQMTQSPSTLSVSVGDR (+19.75 ppm)

Acyl-CoA-binding protein OS=Homo sapiens GN=DBI PE=1 SV=2 - [ACBP_HUMAN]

qATVGDINTERPGmLDFTGk (+3.04 ppm)

Guanine nucleotide-binding protein G(I)/G(S)/G(O) subunit gamma-12 OS=Homo sapiens GN=GNG12 PE=1 SV=3 - [GBG12_HUMAN]

sDPLLIGIPTSENPFk (+1.97 ppm)

Ig lambda chain V-I region WAH OS=Homo sapiens PE=1 SV=1 - [LV106_HUMAN]

dNQRPSGVPDR (+6.47 ppm)

Ig kappa chain V-II region MIL OS=Homo sapiens PE=1 SV=1 - [KV203_HUMAN]

dIVLTQSPLSLPVTPGEPASIScR (+4.03 ppm)

Sodium/potassium-transporting ATPase subunit gamma OS=Homo sapiens GN=FXYD2 PE=1 SV=3 - [ATNG_HUMAN]

gDVDPFYYDYETVR (+10.27 ppm)

Glutaredoxin-1 OS=Homo sapiens GN=GLRX PE=1 SV=2 - [GLRX1_HUMAN]

dcIGGcSDLVSLQQSGELLTR (+0.88 ppm)

Trefoil factor 1 OS=Homo sapiens GN=TFF1 PE=1 SV=1 - [TFF1_HUMAN]

qNcGFPGVTPSQcANk (+3.04 ppm)

Cystatin-A OS=Homo sapiens GN=CSTA PE=1 SV=1 - [CYTA_HUMAN]

sLPGQNEDLVLTGYQVDk (+7.58 ppm)

Complement C4-B OS=Homo sapiens GN=C4B PE=1 SV=2 - [CO4B_HUMAN]

lQETSNWLLSQQQADGSFQDLSPVIHR (+8.17 ppm)

Complement C4-A OS=Homo sapiens GN=C4A PE=1 SV=2 - [CO4A_HUMAN]

lQETSNWLLSQQQADGSFQDPcPVLDR (-0.5 ppm)

Ig kappa chain V-III region CLL OS=Homo sapiens PE=1 SV=2 - [KV308_HUMAN]

fSGSGSGTEFTLTISR (+2.92 ppm)

Ig lambda chain V-IV region Hil OS=Homo sapiens PE=1 SV=1 - [LV403_HUMAN]

sYELTQPPSVSVSPGQTAR (-2.24 ppm)

Ig kappa chain V-I region Wes OS=Homo sapiens PE=1 SV=1 - [KV119_HUMAN]

dIQmTQSPSSVSASVGDR (+4.77 ppm)

Ig kappa chain V-I region BAN OS=Homo sapiens PE=1 SV=1 - [KV122_HUMAN]

dIQLTQSPSSLSASVGDR (-29.55 ppm)

Ig heavy chain V-III region TIL OS=Homo sapiens PE=1 SV=1 - [HV304_HUMAN]

eVQLLESGGGLVQPGGSLR (+3.39 ppm)

Ig kappa chain V-I region HK101 (Fragment) OS=Homo sapiens PE=4 SV=1 - [KV109_HUMAN]

cDIQMTQSPSSLSASVGDR (+45.48 ppm)

Ig heavy chain V-III region TEI OS=Homo sapiens PE=1 SV=1 - [HV316_HUMAN]

eVQLVESGGGLVQPGGSLR (+0.8 ppm)

Thymosin beta-4 OS=Homo sapiens GN=TMSB4X PE=1 SV=2 - [TYB4_HUMAN]

eTIEQEk (+19.25 ppm)

Ig kappa chain V-III region VH (Fragment) OS=Homo sapiens PE=4 SV=1 - [KV310_HUMAN]

eIVMTQSPPTLSLSPGER (9.47 ppm)

UPF0669 protein C6orf120 OS=Homo sapiens GN=C6orf120 PE=1 SV=1 - [CF120_HUMAN]

vYYDGTVEQHPFGEAAYPADGADAGQk (-0.06 ppm)

Programmed cell death protein 6 OS=Homo sapiens GN=PDCD6 PE=1 SV=1 - [PDCD6_HUMAN]

sGVISDTELQQALSNGTWTPFNPVTVR (-0.84 ppm)

Prostate stem cell antigen OS=Homo sapiens GN=PSCA PE=1 SV=1 - [PSCA_HUMAN]

gcSLNcVDDSQDYYVGk (-0.9 ppm)

Molybdopterin synthase sulfur carrier subunit OS=Homo sapiens GN=MOCS2 PE=1 SV=1 - [MOC2A_HUMAN]

sETISVPQEIk (+0.94 ppm)

Hemoglobin subunit epsilon OS=Homo sapiens GN=HBE1 PE=1 SV=2 - [HBE_HUMAN]

lSELHcDk (-8.8 ppm)

Alpha-endosulfine OS=Homo sapiens GN=ENSA PE=1 SV=1 - [ENSA_HUMAN]

qEEENPAEETGEEk (-7.3 ppm)

Proline-rich protein 4 OS=Homo sapiens GN=PRR4 PE=1 SV=3 - [PROL4_HUMAN]

hPPPPPFQNQQRPPR (+6.5 ppm)

Uncharacterized protein C5orf55 OS=Homo sapiens GN=C5orf55 PE=4 SV=1 - [CE055_HUMAN]

vGNQTSVIPATSR (-7.55 ppm)

Interleukin-1 receptor antagonist protein OS=Homo sapiens GN=IL1RN PE=1 SV=1 - [IL1RA_HUMAN]

nNQLVAGYLQGPNVNLEEk (+8.22 ppm)

Neutrophil defensin 4 OS=Homo sapiens GN=DEFA4 PE=1 SV=2 - [DEF4_HUMAN]

gDEAPGQEQR (+7.5 ppm)

Psoriasis susceptibility 1 candidate gene 2 protein OS=Homo sapiens GN=PSORS1C2 PE=2 SV=2 - [PS1C2_HUMAN]

dLPETGVWLPEPPR (+1.76 ppm)

Tachykinin-3 OS=Homo sapiens GN=TAC3 PE=1 SV=1 - [TKNK_HUMAN]

ePQEEVVPGGGR (+5.69 ppm)

2'-deoxynucleoside 5'-phosphate N-hydrolase 1 OS=Homo sapiens GN=DNPH1 PE=1 SV=1 - [DNPH1_HUMAN]

fGTVLTEHVAAAELGAR (+0.97 ppm)

Neutrophil defensin 1 OS=Homo sapiens GN=DEFA1 PE=1 SV=1 - [DEF1_HUMAN]

iPAcIAGER (+16.23 ppm)

Protein CCSMST1 OS=Homo sapiens GN=CCSMST1 PE=2 SV=1 - [CSMT1_HUMAN]

qVWGEVPEPSDR (-14.23 ppm)

Ribonuclease 7 OS=Homo sapiens GN=RNASE7 PE=1 SV=2 - [RNAS7_HUMAN]

dSQQFHLVPVHLDR (-0.56 ppm)

Neuroendocrine protein 7B2 OS=Homo sapiens GN=SCG5 PE=1 SV=2 - [7B2_HUMAN]

dFSEDQGYPDPPNPcPVGk (+10.14 ppm)

Immunoglobulin lambda-like polypeptide 1 OS=Homo sapiens GN=IGLL1 PE=1 SV=1 - [IGLL1_HUMAN]

aTPSVTLFPPSSEELQANk (-3.54 ppm)

UPF0556 protein C19orf10 OS=Homo sapiens GN=C19orf10 PE=1 SV=1 - [CS010_HUMAN]

eSDVPLkTEEFEVTk (+6.6 ppm)

V-set and transmembrane domain-containing protein 1 OS=Homo sapiens GN=VSTM1 PE=1 SV=2 - [VSTM1_HUMAN]

qEQSSAENEAEFPFTDLkPk (-5.73 ppm)

Peroxiredoxin-5, mitochondrial OS=Homo sapiens GN=PRDX5 PE=1 SV=4 - [PRDX5_HUMAN]

vGDAIPAVEVFEGEPGNk (+2.59 ppm)

Charged multivesicular body protein 5 OS=Homo sapiens GN=CHMP5 PE=1 SV=1 - [CHMP5_HUMAN]

nkDGVLVDEFGLPQIPAS (+5.02 ppm)

Ubiquitin carboxyl-terminal hydrolase isozyme L1 OS=Homo sapiens GN=UCHL1 PE=1 SV=2 - [UCHL1_HUMAN]

nEAIQAAHDAVAQEGQcR (-0.04 ppm)

Teratocarcinoma-derived growth factor 1 OS=Homo sapiens GN=TDGF1 PE=1 SV=1 - [TDGF1_HUMAN]

dDSIWPQEEPAIRPR (+2.96 ppm)

Keratin, type II cytoskeletal 8 OS=Homo sapiens GN=KRT8 PE=1 SV=7 - [K2C8_HUMAN]

sLDMDSIIAEVk (+16.16 ppm)

Neurotrimin OS=Homo sapiens GN=NTM PE=1 SV=1 - [NTRI_HUMAN]

gTLQcEASAVPSAEFQWYk (+5.09 ppm)

Protein CutA OS=Homo sapiens GN=CUTA PE=1 SV=2 - [CUTA_HUMAN]

tQSSLVPALTDFVR (+6.43 ppm)

Ig lambda chain V region 4A OS=Homo sapiens PE=4 SV=1 - [LV001_HUMAN]

fSGSLLGGk (-1.93 ppm)

Intraflagellar transport protein 25 homolog OS=Homo sapiens GN=HSPB11 PE=1 SV=1 - [IFT25_HUMAN]

ePVDFEQWIEk (+7.48 ppm)

Protein S100-A12 OS=Homo sapiens GN=S100A12 PE=1 SV=2 - [S10AC_HUMAN]

eLANTIk (-12.75 ppm)

Aquaporin-1 OS=Homo sapiens GN=AQP1 PE=1 SV=3 - [AQP1_HUMAN]

vWTSGQVEEYDLDADDINSR (+6.35 ppm)

Retinoic acid receptor responder protein 2 OS=Homo sapiens GN=RARRES2 PE=1 SV=1 - [RARR2_HUMAN]

eAEEHQETQcLR (+5.93 ppm)

Chondrolectin OS=Homo sapiens GN=CHODL PE=2 SV=2 - [CHODL_HUMAN]

lAcESEGGVLLSLENEAEQk (+13.13 ppm)

Putative nucleoside diphosphate kinase OS=Homo sapiens GN=NME2P1 PE=5 SV=1 - [NDK8_HUMAN]

dRPFFPGLVk (+6.7 ppm)

Chloride intracellular channel protein 1 OS=Homo sapiens GN=CLIC1 PE=1 SV=4 - [CLIC1_HUMAN]

fLDGNELTLADcNLLPk (-9.41 ppm)

Lymphocyte antigen 6D OS=Homo sapiens GN=LY6D PE=1 SV=1 - [LY6D_HUMAN]

tTNTVEPLR (+2.32 ppm)

Beta-1,3-N-acetylglucosaminyltransferase lunatic fringe OS=Homo sapiens GN=LFNG PE=1 SV=2 - [LFNG_HUMAN]

sLAGPAGAAPAPGLGAAAAAPGALVR (+5.39 ppm)

Ig lambda chain V-I region NEWM OS=Homo sapiens PE=1 SV=1 - [LV105_HUMAN]

vFGGGTk (+4.9 ppm)

Matrix Gla protein OS=Homo sapiens GN=MGP PE=1 SV=2 - [MGP_HUMAN]

eAcDDYR (+3.96 ppm)

Peptidyl-prolyl cis-trans isomerase A OS=Homo sapiens GN=PPIA PE=1 SV=2 - [PPIA_HUMAN]

eGMNIVEAMER (+1.2 ppm)

Protein S100-A11 OS=Homo sapiens GN=S100A11 PE=1 SV=2 - [S10AB_HUMAN]

dPGVLDR (-6.41 ppm)

B- and T-lymphocyte attenuator OS=Homo sapiens GN=BTLA PE=1 SV=3 - [BTLA_HUMAN]

qSEHSILAGDPFELEcPVk (-1.53 ppm)

N-acetylglucosamine-1-phosphotransferase subunit gamma OS=Homo sapiens GN=GNPTG PE=1 SV=1 - [GNPTG_HUMAN]

vVEEPNAFGVNNPFLPQASR (-1.53 ppm)

HLA class II histocompatibility antigen gamma chain OS=Homo sapiens GN=CD74 PE=1 SV=3 - [HG2A_HUMAN]

cQEEVSHIPAVHPGSFRPk (+3.28 ppm)

SLAM family member 5 OS=Homo sapiens GN=CD84 PE=1 SV=1 - [SLAF5_HUMAN]

tSVAYVTPGDSETAPVVTVTHR (+3.25 ppm)

Proline-rich protein 24 OS=Homo sapiens GN=PRR24 PE=4 SV=1 - [PRR24_HUMAN]

eTPEAAEGR (+0.6 ppm)

Alpha-crystallin B chain OS=Homo sapiens GN=CRYAB PE=1 SV=2 - [CRYAB_HUMAN]

eEkPAVTAAPk (+1.51 ppm)

F-actin-capping protein subunit alpha-1 OS=Homo sapiens GN=CAPZA1 PE=1 SV=3 - [CAZA1_HUMAN]

fITHAPPGEFNEVFNDVR (-5.62 ppm)

Tumor necrosis factor receptor superfamily member 12A OS=Homo sapiens GN=TNFRSF12A PE=1 SV=1 - [TNR12_HUMAN]

cMDcAScR (-1.74 ppm)

CD177 antigen OS=Homo sapiens GN=CD177 PE=1 SV=2 - [CD177_HUMAN]

qEDFcNNLVNSLPLWAPQPPADPGSLR (+5.42 ppm)

Peptidyl-prolyl cis-trans isomerase C OS=Homo sapiens GN=PPIC PE=1 SV=1 - [PPIC_HUMAN]

tVENFVALATGEk (+7.9 ppm)

Asialoglycoprotein receptor 2 OS=Homo sapiens GN=ASGR2 PE=1 SV=2 - [ASGR2_HUMAN]

ycQLENAHLVVINSWEEQk (-2.94 ppm)

CMRF35-like molecule 8 OS=Homo sapiens GN=CD300A PE=1 SV=2 - [CLM8_HUMAN]

tVAGPVGGSLSVQcPYEk (-3.52 ppm)

Ras-related protein Rab-10 OS=Homo sapiens GN=RAB10 PE=1 SV=1 - [RAB10_HUMAN]

nIDEHANEDVER (+45.76 ppm)

ADM OS=Homo sapiens GN=ADM PE=1 SV=1 - [ADML_HUMAN]

sPEDSSPDAAR (-0.69 ppm)

Decorin OS=Homo sapiens GN=DCN PE=1 SV=1 - [PGS2_HUMAN]

dFEPSLGPVcPFR (+8.13 ppm)

Radixin OS=Homo sapiens GN=RDX PE=1 SV=1 - [RADI_HUMAN]

nQEQLAAELAEFTAk (+11.77 ppm)

N(G),N(G)-dimethylarginine dimethylaminohydrolase 1 OS=Homo sapiens GN=DDAH1 PE=1 SV=3 - [DDAH1_HUMAN]

dYAVSTVPVADGLHLk (+12.45 ppm)

C-X-C motif chemokine 16 OS=Homo sapiens GN=CXCL16 PE=2 SV=4 - [CXL16_HUMAN]

ecGHAYSGIVAHQk (+2.67 ppm)

Histone H2A type 1-H OS=Homo sapiens GN=HIST1H2AH PE=1 SV=3 - [H2A1H_HUMAN]

nDEELNk (+2.79 ppm)

CD99 antigen OS=Homo sapiens GN=CD99 PE=1 SV=1 - [CD99_HUMAN]

gGSDGGGSHR (+5.65 ppm)

N-acetyllactosaminide beta-1,3-N-acetylglucosaminyltransferase OS=Homo sapiens GN=B3GNT1 PE=1 SV=1 - [B3GN1_HUMAN]

eMLDQSNQWGGTALVVPAFEIR (+5.11 ppm)

Histone H3.1 OS=Homo sapiens GN=HIST1H3A PE=1 SV=2 - [H31_HUMAN]

eIAQDFk (+10.5 ppm)

Carbonic anhydrase 12 OS=Homo sapiens GN=CA12 PE=1 SV=1 - [CAH12_HUMAN]

gQEAFVPGFNIEELLPER (+2.21 ppm)

Putative uncharacterized protein CYorf17 OS=Homo sapiens GN=CYorf17 PE=2 SV=1 - [CY017_HUMAN]

dPESLLFLR (-13.61 ppm)

Iduronate 2-sulfatase OS=Homo sapiens GN=IDS PE=1 SV=1 - [IDS_HUMAN]

gPDGELHANLLcPVDVLDVPEGTLPDk (+2.88 ppm)

Keratinocyte-associated transmembrane protein 2 OS=Homo sapiens GN=KCT2 PE=2 SV=2 - [KCT2_HUMAN]

dDDESDDTLEENR (+1.16 ppm)

Myelin protein zero-like protein 1 OS=Homo sapiens GN=MPZL1 PE=1 SV=1 - [MPZL1_HUMAN]

nPPDIVVQPGHIR (+6.66 ppm)

Cofilin-1 OS=Homo sapiens GN=CFL1 PE=1 SV=3 - [COF1_HUMAN]

nIILEEGk (-11.19 ppm)

Lymphocyte function-associated antigen 3 OS=Homo sapiens GN=CD58 PE=1 SV=1 - [LFA3_HUMAN]

dkVAELENSEFR (+20.18 ppm)

IGF-like family receptor 1 OS=Homo sapiens GN=IGFLR1 PE=1 SV=1 - [IGFR1_HUMAN]

eNcGLNDHGDFVTPPFR (+4.95 ppm)

Programmed cell death 1 ligand 2 OS=Homo sapiens GN=PDCD1LG2 PE=1 SV=2 - [PD1L2_HUMAN]

tPEGLYQVTSVLR (+4.14 ppm)

Augurin OS=Homo sapiens GN=C2orf40 PE=1 SV=1 - [AUGN_HUMAN]

eFLGSLk (+4.61 ppm)

Ras-related protein Rap-1b-like protein OS=Homo sapiens PE=2 SV=1 - [RP1BL_HUMAN]

eQGQNLAR (+3.59 ppm)

Synaptic vesicle membrane protein VAT-1 homolog OS=Homo sapiens GN=VAT1 PE=1 SV=2 - [VAT1_HUMAN]

eVAEAATGEDASSPPPk (-6.25 ppm)

Endothelin-3 OS=Homo sapiens GN=EDN3 PE=1 SV=1 - [EDN3_HUMAN]

gVSQAPTAAR (+1.05 ppm)

Keratin, type II cytoskeletal 72 OS=Homo sapiens GN=KRT72 PE=1 SV=2 - [K2C72_HUMAN]

fLEQQNQVLETk (+8.69 ppm)

Tubulin beta chain OS=Homo sapiens GN=TUBB PE=1 SV=2 - [TBB5_HUMAN]

gHYTEGAELVDSVLDVVR (+4.91 ppm)

Plexin domain-containing protein 1 OS=Homo sapiens GN=PLXDC1 PE=1 SV=2 - [PLDX1_HUMAN]

tGLSDAFmILNPSPDVPESR (-30.39 ppm)

Ammonium transporter Rh type C OS=Homo sapiens GN=RHCG PE=1 SV=1 - [RHCG_HUMAN]

eGLVHSFDFQGFNGDWTAR (+2.32 ppm)

Legumain OS=Homo sapiens GN=LGMN PE=1 SV=1 - [LGMN_HUMAN]

dYTGEDVTPQNFLAVLR (-0.2 ppm)

V-set domain-containing T-cell activation inhibitor 1 OS=Homo sapiens GN=VTCN1 PE=1 SV=1 - [VTCN1_HUMAN]

eGVLGLVHEFk (+11.72 ppm)

Malate dehydrogenase, cytoplasmic OS=Homo sapiens GN=MDH1 PE=1 SV=4 - [MDHC_HUMAN]

dVIATDkEDVAFk (+10.91 ppm)

Pituitary tumor-transforming gene 1 protein-interacting protein OS=Homo sapiens GN=PTTG1IP PE=1 SV=1 - [PTTG_HUMAN]

tcEEcLk (-4.31 ppm)

Pro-cathepsin H OS=Homo sapiens GN=CTSH PE=1 SV=4 - [CATH_HUMAN]

vNHAVLAVGYGEk (+5.36 ppm)

Asialoglycoprotein receptor 1 OS=Homo sapiens GN=ASGR1 PE=1 SV=2 - [ASGR1_HUMAN]

gLSTQGGNVGR (+8.82 ppm)

Nucleoredoxin-like protein 1 OS=Homo sapiens GN=NXNL1 PE=2 SV=1 - [NXNL1_HUMAN]

dGADEIQR (+4.91 ppm)

WD repeat-containing protein 1 OS=Homo sapiens GN=WDR1 PE=1 SV=4 - [WDR1_HUMAN]

nIDNPALADIYTEHAHQVVVAk (+11.6 ppm)

Arylsulfatase B OS=Homo sapiens GN=ARSB PE=1 SV=1 - [ARSB_HUMAN]

iELLHNIDPNFVDSSPcPR (+3.77 ppm)

Osteoclast-associated immunoglobulin-like receptor OS=Homo sapiens GN=OSCAR PE=2 SV=3 - [OSCAR_HUMAN]

eGVAAPLQYR (+3.34 ppm)

CMRF35-like molecule 2 OS=Homo sapiens GN=CD300E PE=1 SV=2 - [CLM2_HUMAN]

dPSDLVR (-0.21 ppm)

Ubiquilin-1 OS=Homo sapiens GN=UBQLN1 PE=1 SV=2 - [UBQL1_HUMAN]

eANLQALIATGGDINAAIER (+6.25 ppm)

Matrilysin OS=Homo sapiens GN=MMP7 PE=1 SV=1 - [MMP7_HUMAN]

dLPHITVDR (+1.08 ppm)

Putative alpha-1-antitrypsin-related protein OS=Homo sapiens GN=SERPINA2 PE=1 SV=1 - [A1ATR_HUMAN]

mLSLGTk (+3.53 ppm)

Xaa-Pro dipeptidase OS=Homo sapiens GN=PEPD PE=1 SV=3 - [PEPD_HUMAN]

fEVNNTILHPEIVEcR (+11.29 ppm)

NSFL1 cofactor p47 OS=Homo sapiens GN=NSFL1C PE=1 SV=2 - [NSF1C_HUMAN]

eFVAVTGAEEDR (+2.78 ppm)

Transmembrane gamma-carboxyglutamic acid protein 1 OS=Homo sapiens GN=PRRG1 PE=1 SV=1 - [TMG1_HUMAN]

sDSVSTR (+12.15 ppm)

Intelectin-1 OS=Homo sapiens GN=ITLN1 PE=1 SV=1 - [ITLN1_HUMAN]

dLGIWHVPNk (+11.7 ppm)

Plastin-3 OS=Homo sapiens GN=PLS3 PE=1 SV=4 - [PLST_HUMAN]

tLSEAGk (+5.21 ppm)

Vasodilator-stimulated phosphoprotein OS=Homo sapiens GN=VASP PE=1 SV=3 - [VASP_HUMAN]

dESANQEEPEAR (+5.88 ppm)

Poliovirus receptor OS=Homo sapiens GN=PVR PE=1 SV=2 - [PVR_HUMAN]

vLAkPQNTAEVQk (+1.82 ppm)

Glypican-3 OS=Homo sapiens GN=GPC3 PE=1 SV=1 - [GPC3_HUMAN]

wVPETPVPGSDLQVcLPk (+15.23 ppm)

Sodium/nucleoside cotransporter 1 OS=Homo sapiens GN=SLC28A1 PE=1 SV=2 - [S28A1_HUMAN]

eAFQSVNPEFSPEALDNccR (-3.48 ppm)

Cytochrome c oxidase subunit 2 OS=Homo sapiens GN=MT-CO2 PE=1 SV=1 - [COX2_HUMAN]

tDAIPGR (+4.81 ppm)

CD276 antigen OS=Homo sapiens GN=CD276 PE=1 SV=1 - [CD276_HUMAN]

dLRPGDTVTITcSSYR (+5.92 ppm)

G-protein coupled receptor family C group 5 member B OS=Homo sapiens GN=GPRC5B PE=2 SV=2 - [GPC5B_HUMAN]

eTAFEEDVQLPR (+16.96 ppm)

Lysosomal thioesterase PPT2 OS=Homo sapiens GN=PPT2 PE=1 SV=4 - [PPT2_HUMAN]

eAVVPIMAk (+2.07 ppm)

Protein-glutamine gamma-glutamyltransferase 4 OS=Homo sapiens GN=TGM4 PE=1 SV=2 - [TGM4_HUMAN]

nVLDccISLLTESSLkPTDR (+10.87 ppm)

Protein YIPF3 OS=Homo sapiens GN=YIPF3 PE=1 SV=1 - [YIPF3_HUMAN]

dIPAmLPAAR (+2.79 ppm)

Otoconin-90 OS=Homo sapiens GN=OC90 PE=2 SV=3 - [OC90_HUMAN]

lPWSPVVcVDHTPk (+5.11 ppm)

Amphiphysin OS=Homo sapiens GN=AMPH PE=1 SV=1 - [AMPH_HUMAN]

vETLHDFEAANSDELTLQR (+3.96 ppm)

GDNF family receptor alpha-1 OS=Homo sapiens GN=GFRA1 PE=2 SV=2 - [GFRA1_HUMAN]

eGLGASSHITTk (+2.33 ppm)

Delta and Notch-like epidermal growth factor-related receptor OS=Homo sapiens GN=DNER PE=1 SV=1 - [DNER_HUMAN]

vSTcVPGESHANDLEcSGk (-1.06 ppm)

Protocadherin-18 OS=Homo sapiens GN=PCDH18 PE=2 SV=3 - [PCD18_HUMAN]

fRAMQRGNSPLLVVNEDNGEISIGATIDR (-14.3 ppm)

Thrombospondin-2 OS=Homo sapiens GN=THBS2 PE=1 SV=2 - [TSP2_HUMAN]

dGIGDAcDDDDDNDGVTDEkDNcQLLFNPR

Cadherin-16 OS=Homo sapiens GN=CDH16 PE=1 SV=1 - [CAD16_HUMAN]

fHILSQAPAQPSPDmFQLEPR (+7.99 ppm)

Uncharacterized protein KIAA1211-like OS=Homo sapiens GN=KIAA1211L PE=2 SV=3 - [K121L_HUMAN]

sLPPAATGPGADGQPAPPWITVTR (+20.14 ppm)

Plexin domain-containing protein 2 OS=Homo sapiens GN=PLXDC2 PE=1 SV=1 - [PXDC2_HUMAN]dNGASTDDSAAEk (+1.37 ppm)

Alcohol dehydrogenase [NADP(+)] OS=Homo sapiens GN=AKR1A1 PE=1 SV=3 - [AK1A1_HUMAN]

eELFVTSk (+23.42 ppm)

Cysteine-rich secretory protein LCCL domain-containing 2 OS=Homo sapiens GN=CRISPLD2 PE=1 SV=1 - [CRLD2_HUMAN]

eDkEEILmLHNk (-0.12 ppm)

Protein shisa-6 homolog OS=Homo sapiens GN=SHISA6 PE=2 SV=2 - [SHSA6_HUMAN]

tLSAGGAAVGGR (+9.91 ppm)

Protein FAM151A OS=Homo sapiens GN=FAM151A PE=2 SV=2 - [F151A_HUMAN]

dALEVTWYHAANSk (+2.61 ppm)

Protein shisa-7 OS=Homo sapiens GN=SHISA7 PE=2 SV=3 - [SHSA7_HUMAN]

tGPAGGAGAAAR (-3.5 ppm)

Mesothelin OS=Homo sapiens GN=MSLN PE=1 SV=2 - [MSLN_HUMAN]

tDAVLPLTVAEVQk (+5.29 ppm)

PDZ domain-containing RING finger protein 4 OS=Homo sapiens GN=PDZRN4 PE=1 SV=3 - [PZRN4_HUMAN]

iLDNWMTIQELmTHGAkSPDGTR (+17.08 ppm)

TGF-beta receptor type-1 OS=Homo sapiens GN=TGFBR1 PE=1 SV=1 - [TGFR1_HUMAN]

dRPFVcAPSSk (+6.93 ppm)

Protein phosphatase 1 regulatory subunit 37 OS=Homo sapiens GN=PPP1R37 PE=1 SV=4 - [PPR37_HUMAN]

dTGSSEPQPPPEPPR (+46.91 ppm)

Secretogranin-2 OS=Homo sapiens GN=SCG2 PE=1 SV=2 - [SCG2_HUMAN]

eHLNQGSSQETDk (+15.49 ppm)

Protein delta homolog 1 OS=Homo sapiens GN=DLK1 PE=1 SV=3 - [DLK1_HUMAN]

cPAGFIDk (-6.14 ppm)

Major vault protein OS=Homo sapiens GN=MVP PE=1 SV=4 - [MVP_HUMAN]

vVAGDEWLFEGPGTYIPR (+3.19 ppm)

Sorting nexin-9 OS=Homo sapiens GN=SNX9 PE=1 SV=1 - [SNX9_HUMAN]

dSESADAGGAQR (+6.82 ppm)

Transforming growth factor beta receptor type 3 OS=Homo sapiens GN=TGFBR3 PE=1 SV=3 - [TGBR3_HUMAN]

iLLDPGALPALQNPPIR (+2.73 ppm)

Cochlin OS=Homo sapiens GN=COCH PE=1 SV=1 - [COCH_HUMAN]

gVISNSGGPVR (+0.64 ppm)

Angiotensin-converting enzyme 2 OS=Homo sapiens GN=ACE2 PE=1 SV=2 - [ACE2_HUMAN]

tLYQFQFQEALcQAAk (-2.87 ppm)

UV excision repair protein RAD23 homolog B OS=Homo sapiens GN=RAD23B PE=1 SV=1 - [RD23B_HUMAN]

eQVIAALR (+3.66 ppm)

Heterogeneous nuclear ribonucleoprotein K OS=Homo sapiens GN=HNRNPK PE=1 SV=1 - [HNRPK_HUMAN]

dLAGSIIGk (-12.3 ppm)

Anosmin-1 OS=Homo sapiens GN=KAL1 PE=1 SV=3 - [KALM_HUMAN]

tTDGFQNSVILEk (+31.17 ppm)

Desmoglein-2 OS=Homo sapiens GN=DSG2 PE=1 SV=2 - [DSG2_HUMAN]

gQIIGNFQAFDEDTGLPAHAR (+8.19 ppm)

Frizzled-8 OS=Homo sapiens GN=FZD8 PE=1 SV=1 - [FZD8_HUMAN]

eLAcQEITVPLck (+3.81 ppm)

Complement component C8 beta chain OS=Homo sapiens GN=C8B PE=1 SV=3 - [CO8B_HUMAN]

cEGFVcAQTGR (-0.96 ppm)

Calsyntenin-1 OS=Homo sapiens GN=CLSTN1 PE=1 SV=1 - [CSTN1_HUMAN]

aASEFESSEGVFLFPELR (+4.6 ppm)

Desmoglein-1 OS=Homo sapiens GN=DSG1 PE=1 SV=2 - [DSG1_HUMAN]

vGDFVATDLDTGRPSTTVR (-2.5 ppm)

Phosphofurin acidic cluster sorting protein 1 OS=Homo sapiens GN=PACS1 PE=1 SV=2 - [PACS1_HUMAN]

gGAGGGPGGAGGGSGQR (+1.03 ppm)

Seizure 6-like protein 2 OS=Homo sapiens GN=SEZ6L2 PE=1 SV=2 - [SE6L2_HUMAN]

eGDMLTLFDGDGPSAR (-0.78 ppm)

Solute carrier family 12 member 3 OS=Homo sapiens GN=SLC12A3 PE=1 SV=3 - [S12A3_HUMAN]

dLLQEYGAPIVDPINDIR (+13.79 ppm)

Extracellular serine/threonine protein kinase FAM20C OS=Homo sapiens GN=FAM20C PE=1 SV=2 - [DMP4_HUMAN]

lPPAAEPAER (+5.56 ppm)

Amiloride-sensitive sodium channel subunit gamma OS=Homo sapiens GN=SCNN1G PE=1 SV=4 - [SCNNG_HUMAN]

iPLLIFDQDEk (-10.31 ppm)

Carboxypeptidase Z OS=Homo sapiens GN=CPZ PE=1 SV=2 - [CBPZ_HUMAN]

eDEGcYDPLEk (-0.6 ppm)

Hydroperoxide isomerase ALOXE3 OS=Homo sapiens GN=ALOXE3 PE=1 SV=1 - [LOXE3_HUMAN]

cTAELGELLLLR (-0.16 ppm)

Copine-3 OS=Homo sapiens GN=CPNE3 PE=1 SV=1 - [CPNE3_HUMAN]

dIVQFVPFR (+3.53 ppm)

Atrial natriuretic peptide receptor 3 OS=Homo sapiens GN=NPR3 PE=1 SV=2 - [ANPRC_HUMAN]

dLDLEDIVR (+3.31 ppm)

EH domain-binding protein 1-like protein 1 OS=Homo sapiens GN=EHBP1L1 PE=1 SV=2 - [EH1L1_HUMAN]

eSRPAEVPAEGLVNGAGAPGGGGVR (-0.96 ppm)

Protein crumbs homolog 2 OS=Homo sapiens GN=CRB2 PE=1 SV=2 - [CRUM2_HUMAN]

vDGHLLLPEDLGENVLLGcER (-2.76 ppm)

Aldehyde dehydrogenase, mitochondrial OS=Homo sapiens GN=ALDH2 PE=1 SV=2 - [ALDH2_HUMAN]

tIEEVVGR (+6.54 ppm)

Receptor-type tyrosine-protein phosphatase delta OS=Homo sapiens GN=PTPRD PE=1 SV=2 - [PTPRD_HUMAN]

dGEHGEEQR (-0.84 ppm)

Pre-mRNA-splicing regulator WTAP OS=Homo sapiens GN=WTAP PE=1 SV=2 - [FL2D_HUMAN]

dELILR (+0.12 ppm)

Extracellular sulfatase Sulf-2 OS=Homo sapiens GN=SULF2 PE=1 SV=1 - [SULF2_HUMAN]

vYHVGLGDAAQPR (4.42 ppm)

T-cell immunomodulatory protein OS=Homo sapiens GN=ITFG1 PE=1 SV=1 - [TIP_HUMAN]

nDFAIHTLk (+11.12 ppm)

Collagen alpha-1(XXVIII) chain OS=Homo sapiens GN=COL28A1 PE=2 SV=2 - [COSA1_HUMAN]

cLEALkPGNcGEYVVR (+9.85 ppm)

Calsyntenin-3 OS=Homo sapiens GN=CLSTN3 PE=1 SV=1 - [CSTN3_HUMAN]

eSLLLDTTSLQQR (+14.14 ppm)

Zinc finger and BTB domain-containing protein 18 OS=Homo sapiens GN=ZBTB18 PE=1 SV=1 - [ZBT18_HUMAN]

lNILPSk (+3.82 ppm)

Asparagine--tRNA ligase, cytoplasmic OS=Homo sapiens GN=NARS PE=1 SV=1 - [SYNC_HUMAN]

nLEEAkk (+9.26 ppm)

Tetratricopeptide repeat protein 26 OS=Homo sapiens GN=TTC26 PE=2 SV=1 - [TTC26_HUMAN]

lEELLSk (+2.68 ppm)

Bone morphogenetic protein receptor type-2 OS=Homo sapiens GN=BMPR2 PE=1 SV=2 - [BMPR2_HUMAN]

dPYQQDLGIGESR (+1.08 ppm)

Kinetochore protein NDC80 homolog OS=Homo sapiens GN=NDC80 PE=1 SV=1 - [NDC80_HUMAN]

hLEEQIAk (+12.04 ppm)

Myosin-8 OS=Homo sapiens GN=MYH8 PE=1 SV=3 - [MYH8_HUMAN]

qREEQAEPDGTEVADk (-6.51 ppm)

Lipolysis-stimulated lipoprotein receptor OS=Homo sapiens GN=LSR PE=1 SV=4 - [LSR_HUMAN]

sPTSNGGR (-0.04 ppm)

Complement component C1q receptor OS=Homo sapiens GN=CD93 PE=1 SV=3 - [C1QR1_HUMAN]

eEAQHVQR (+6.18 ppm)

Phosphorylase b kinase regulatory subunit alpha, skeletal muscle isoform OS=Homo sapiens GN=PHKA1 PE=1 SV=2 - [KPB1_HUMAN]

lDGALNRVPVGFYQk (-9.68 ppm)

Brevican core protein OS=Homo sapiens GN=BCAN PE=1 SV=2 - [PGCB_HUMAN]

aLHPEEDPEGR (+5.52 ppm)

PCNA-interacting partner OS=Homo sapiens GN=PARPBP PE=1 SV=3 - [PARI_HUMAN]

tIELGGk (-3.96 ppm)

Ras-specific guanine nucleotide-releasing factor RalGPS2 OS=Homo sapiens GN=RALGPS2 PE=1 SV=1 - [RGPS2_HUMAN]

dYISSLk (+14.71 ppm)

Syntaxin-binding protein 2 OS=Homo sapiens GN=STXBP2 PE=1 SV=2 - [STXB2_HUMAN]

dLSQILk (-1 ppm)

Spermatogenesis-associated protein 7 OS=Homo sapiens GN=SPATA7 PE=2 SV=3 - [SPAT7_HUMAN]

aTSVLPR (+1.2 ppm)

Uncharacterized protein C14orf37 OS=Homo sapiens GN=C14orf37 PE=1 SV=1 - [CN037_HUMAN]

eIAHVHAEk (+11.36 ppm)

Endoribonuclease Dicer OS=Homo sapiens GN=DICER1 PE=1 SV=3 - [DICER_HUMAN]

lLmELEEALNFINDcNISVHSk (+1.85 ppm)

PHD finger protein 14 OS=Homo sapiens GN=PHF14 PE=1 SV=2 - [PHF14_HUMAN]

eEEENGERPR (+3.25 ppm)

Integrin alpha-7 OS=Homo sapiens GN=ITGA7 PE=1 SV=3 - [ITA7_HUMAN]

eLEPPEQQEPGER (+3.74 ppm)

Sarcoplasmic reticulum histidine-rich calcium-binding protein OS=Homo sapiens GN=HRC PE=2 SV=1 - [SRCH_HUMAN]

gDGLGFR (+2 ppm)

PH domain leucine-rich repeat-containing protein phosphatase 1 OS=Homo sapiens GN=PHLPP1 PE=1 SV=3 - [PHLP1_HUMAN]

lcMSGNcVETLRLQALR (-2.73 ppm)

Tetratricopeptide repeat protein 21B OS=Homo sapiens GN=TTC21B PE=1 SV=2 - [TT21B_HUMAN]

lLLQDSQNVEALR (-12.23 ppm)

Fibroblast growth factor receptor 1 OS=Homo sapiens GN=FGFR1 PE=1 SV=3 - [FGFR1_HUMAN]

eMEVLHLR (+6.51 ppm)

Biorientation of chromosomes in cell division protein 1-like 1 OS=Homo sapiens GN=BOD1L1 PE=1 SV=2 - [BD1L1_HUMAN]

sGDmTLIPEQEPmEIDSEPGVENVFEVSk (+16.33 ppm)

Multimerin-2 OS=Homo sapiens GN=MMRN2 PE=1 SV=2 - [MMRN2_HUMAN]

eAEPLVDIR (+1.88 ppm)

Zinc finger SWIM domain-containing protein 8 OS=Homo sapiens GN=ZSWIM8 PE=1 SV=1 - [ZSWM8_HUMAN]

tTVSEAEHPLLcEGTRR (+2.02 ppm)

Epidermal growth factor receptor OS=Homo sapiens GN=EGFR PE=1 SV=2 - [EGFR_HUMAN]

nLQEILHGAVR (+9.34 ppm)

ATP-dependent zinc metalloprotease YME1L1 OS=Homo sapiens GN=YME1L1 PE=1 SV=2 - [YMEL1_HUMAN]

sVEIDNk (+11.94 ppm)

Papilin OS=Homo sapiens GN=PAPLN PE=2 SV=4 - [PPN_HUMAN]

dGGSScVGPAR (+4.56 ppm)

Dyslexia-associated protein KIAA0319-like protein OS=Homo sapiens GN=KIAA0319L PE=1 SV=2 - [K319L_HUMAN]

dYSGEMEGk (+0.72 ppm)

A disintegrin and metalloproteinase with thrombospondin motifs 5 OS=Homo sapiens GN=ADAMTS5 PE=1 SV=2 - [ATS5_HUMAN]

qGEEVQER (+10.6 ppm)

VPS10 domain-containing receptor SorCS2 OS=Homo sapiens GN=SORCS2 PE=1 SV=3 - [SORC2_HUMAN]

aEPGGGEDR (+5.97 ppm)

Probable G-protein coupled receptor 116 OS=Homo sapiens GN=GPR116 PE=1 SV=3 - [GP116_HUMAN]

dVIVHPLPLk (+10.22 ppm)

EGF-like module-containing mucin-like hormone receptor-like 1 OS=Homo sapiens GN=EMR1 PE=2 SV=3 - [EMR1_HUMAN]

dIDEcR (+7.3 ppm)

Rho GTPase-activating protein 29 OS=Homo sapiens GN=ARHGAP29 PE=1 SV=2 - [RHG29_HUMAN]

lEEEALQk (+6.52 ppm)

Myelin transcription factor 1-like protein OS=Homo sapiens GN=MYT1L PE=2 SV=3 - [MYT1L_HUMAN]

eTVDSLk (+6.52 ppm)

Collagen alpha-2(V) chain OS=Homo sapiens GN=COL5A2 PE=1 SV=3 - [CO5A2_HUMAN]

dGAVGER (+3.12 ppm)

Thyroid adenoma-associated protein OS=Homo sapiens GN=THADA PE=1 SV=1 - [THADA_HUMAN]

eLIAAELk (+6.21 ppm)

Stabilin-2 OS=Homo sapiens GN=STAB2 PE=1 SV=3 - [STAB2_HUMAN]

nDLHNGmHR (+7.63 ppm)

Leucine-rich repeat and WD repeat-containing protein KIAA1239 OS=Homo sapiens GN=KIAA1239 PE=2 SV=3 - [K1239_HUMAN]

vDAALk (+31.16 ppm)

Histone-lysine N-methyltransferase 2A OS=Homo sapiens GN=KMT2A PE=1 SV=5 - [KMT2A_HUMAN]

qPPPPESGPEQSk (+17.65 ppm)

InaD-like protein OS=Homo sapiens GN=INADL PE=1 SV=3 - [INADL_HUMAN]

iVELVk (+9.33 ppm)

Protein SZT2 OS=Homo sapiens GN=SZT2 PE=2 SV=3 - [SZT2_HUMAN]

qNLLIFLHSPk (

Chromodomain-helicase-DNA-binding protein 8 OS=Homo sapiens GN=CHD8 PE=1 SV=5 - [CHD8_HUMAN]

eIEDLLR (+0.45 ppm)

Mucin-5B OS=Homo sapiens GN=MUC5B PE=1 SV=3 - [MUC5B_HUMAN]

nWEQEGVFk (-6.6 ppm)
