# Supplementary material for: Custom 4-Plex DiLeu Isobaric Labels Enable Relative Quantification of Urinary Proteins in Men with Lower Urinary Tract Symptoms (LUTS)
Source: PLoS One. 2015 Aug 12;10(8):e0135415. doi: 10.1371/journal.pone.0135415 (PMC4534462; doi:10.1371/journal.pone.0135415)
Supplement: S1 Table — (DOCX) [file pone.0135415.s004.docx]

**S1 Table. Differentially expressed proteins determined by DiLeu labeling of LUTS urine.**

| **Uniprot**  **Accession** | **Protein Group Name** | **Gene names (primary )** | **T-test** | | **Average Normalized Intensity in LUTS Patients (A)** | | **Average Normalized Intensity in Control Patients (C)** | **Ratio (A/C)** | **Coefficient of Variation in A** | **Coefficient of Variation in C** | **Unique Peptides** |
| --- | --- | --- | --- | --- | --- | --- | --- | --- | --- | --- | --- |
| P08779 | Keratin, type I cytoskeletal 16 | KRT16 | | 0.00 | | 1.24 | 0.62 | 2.00 | 16.19 | 40.12 | 4 |
| P15151 | Poliovirus receptor | PVR | | 0.04 | | 1.34 | 0.68 | 1.97 | 22.56 | 30.28 | 1 |
| Q02383 | Semenogelin-2 | SEMG2 | | 0.04 | | 1.31 | 0.68 | 1.92 | 19.52 | 10.25 | 11 |
| Q9NP84 | Tumor necrosis factor receptor superfamily member 12A | TNFRSF12A | | 0.00 | | 1.36 | 0.71 | 1.92 | 11.48 | 19.26 | 1 |
| P51170 | Amiloride-sensitive sodium channel subunit gamma | SCNN1G | | 0.05 | | 1.25 | 0.69 | 1.80 | 2.15 | 12.11 | 1 |
| P55259 | Pancreatic secretory granule membrane major glycoprotein | GP2 | | 0.03 | | 1.15 | 0.66 | 1.74 | 23.58 | 27.43 | 2 |
| P23352 | Anosmin-1 | KAL1 | | 0.00 | | 1.22 | 0.74 | 1.65 | 18.60 | 34.06 | 1 |
| P21741 | Midkine | MDK | | 0.04 | | 1.22 | 0.76 | 1.62 | 6.55 | 25.33 | 2 |
| P04279 | Semenogelin-1 | SEMG1 | | 0.03 | | 1.13 | 0.72 | 1.56 | 30.02 | 43.21 | 16 |
| P26447 | Protein S100-A4 | S100A4 | | 0.00 | | 1.12 | 0.73 | 1.53 | 11.11 | 18.77 | 2 |
| P02042 | Hemoglobin subunit delta | HBD | | 0.01 | | 1.09 | 0.71 | 1.53 | 7.21 | 18.86 | 5 |
| P02766 | Transthyretin | TTR | | 0.01 | | 1.13 | 0.76 | 1.50 | 17.01 | 29.00 | 3 |
| P30043 | Flavin reductase (NADPH) | BLVRB | | 0.01 | | 1.06 | 0.72 | 1.47 | 6.70 | 4.17 | 2 |
| Q02509 | Otoconin-90 | OC90 | | 0.01 | | 1.19 | 0.80 | 1.47 | 8.54 | 15.12 | 1 |
| P02533 | Keratin, type I cytoskeletal 14 | KRT14 | | 0.00 | | 1.13 | 0.78 | 1.44 | 11.29 | 17.34 | 4 |
| Q07654 | Trefoil factor 3 | TFF3 | | 0.01 | | 1.13 | 0.79 | 1.43 | 7.45 | 17.20 | 3 |
| P14138 | Endothelin-3 | EDN3 | | 0.02 | | 1.12 | 0.79 | 1.41 | 8.73 | 20.65 | 1 |
| Q9H1Z8 | Augurin | C2orf40 | | 0.00 | | 1.12 | 0.80 | 1.40 | 6.65 | 13.29 | 1 |
| P07108 | Acyl-CoA-binding protein | DBI | | 0.00 | | 1.10 | 0.79 | 1.40 | 5.21 | 20.98 | 1 |
| P01033 | Metalloproteinase inhibitor 1 | TIMP1 | | 0.01 | | 1.13 | 0.82 | 1.39 | 5.89 | 4.21 | 2 |
| P08253 | 72 kDa type IV collagenase | MMP2 | | 0.02 | | 1.12 | 0.81 | 1.38 | 2.73 | 11.21 | 2 |
| P13797 | Plastin-3 | PLS3 | | 0.01 | | 1.13 | 0.82 | 1.38 | 16.39 | 14.78 | 2 |
| Q15007 | Pre-mRNA-splicing regulator | WTAP | | 0.05 | | 1.13 | 0.82 | 1.38 | 2.60 | 7.48 | 1 |
| P04792 | Heat shock protein beta-1 | HSPB1 | | 0.00 | | 1.07 | 0.78 | 1.37 | 5.11 | 15.81 | 2 |
| P00748 | Coagulation factor XII | F12 | | 0.01 | | 1.10 | 0.81 | 1.36 | 7.46 | 16.58 | 3 |
| P07148 | Fatty acid-binding protein, liver | FABP1 | | 0.02 | | 1.17 | 0.86 | 1.35 | 18.44 | 20.76 | 3 |
| P54710 | Sodium/potassium-transporting ATPase subunit gamma | FXYD2 | | 0.01 | | 1.14 | 0.85 | 1.34 | 9.85 | 21.20 | 1 |
| P12277 | Creatine kinase B-type | CKB | | 0.01 | | 1.10 | 0.83 | 1.34 | 10.25 | 21.21 | 2 |
| Q14914 | Prostaglandin reductase 1 | PTGR1 | | 0.02 | | 1.09 | 0.82 | 1.33 | 12.84 | 21.81 | 2 |
| P62937 | Peptidyl-prolyl cis-trans isomerase A | PPIA | | 0.02 | | 1.03 | 0.78 | 1.32 | 5.96 | 16.62 | 1 |
| P05387 | 60S acidic ribosomal protein P2 | RPLP2 | | 0.05 | | 1.04 | 0.80 | 1.30 | 8.10 | 19.27 | 2 |
| P15309 | Prostatic acid phosphatase | ACPP | | 0.03 | | 1.09 | 0.84 | 1.29 | 17.07 | 33.13 | 12 |
| P08697 | Alpha-2-antiplasmin | SERPINF2 | | 0.00 | | 1.08 | 0.83 | 1.29 | 7.49 | 16.04 | 5 |
| P13671 | Complement component C6 | C6 | | 0.02 | | 1.06 | 0.83 | 1.27 | 4.94 | 9.83 | 3 |
| Q99497 | Protein DJ-1 | PARK7 | | 0.00 | | 1.06 | 0.84 | 1.25 | 7.85 | 12.91 | 3 |
| P32119 | Peroxiredoxin-2 | PRDX2 | | 0.05 | | 1.04 | 0.83 | 1.25 | 12.23 | 32.24 | 5 |
| O75094 | Slit homolog 3 protein | SLIT3 | | 0.04 | | 1.08 | 0.87 | 1.24 | 12.54 | 19.10 | 3 |
| P43652 | Afamin | AFM | | 0.00 | | 1.08 | 0.88 | 1.22 | 11.59 | 8.27 | 15 |
| P02792 | Ferritin light chain | FTL | | 0.04 | | 1.06 | 0.87 | 1.21 | 12.12 | 19.25 | 2 |
| Q9BQE3 | Tubulin alpha-1C chain | TUBA1C | | 0.00 | | 1.08 | 0.90 | 1.21 | 7.71 | 10.07 | 7 |
| Q09666 | Neuroblast differentiation-associated protein AHNAK | AHNAK | | 0.04 | | 1.05 | 0.87 | 1.20 | 1.00 | 2.89 | 3 |
| O75531 | Barrier-to-autointegration factor | BANF1 | | 0.04 | | 0.91 | 1.13 | 0.80 | 12.47 | 20.99 | 3 |
| Q9UGM3 | Deleted in malignant brain tumors 1 protein | DMBT1 | | 0.01 | | 0.92 | 1.16 | 0.80 | 10.41 | 19.13 | 2 |
| O75864 | Protein phosphatase 1 regulatory subunit 37 | PPP1R37 | | 0.03 | | 0.90 | 1.13 | 0.80 | 12.44 | 10.30 | 1 |
| Q05707 | Collagen alpha-1(XIV) chain | COL14A1 | | 0.03 | | 0.91 | 1.15 | 0.79 | 2.02 | 7.52 | 2 |
| P00352 | Retinal dehydrogenase 1 | ALDH1A1 | | 0.00 | | 0.91 | 1.16 | 0.78 | 6.96 | 7.41 | 3 |
| P45877 | Peptidyl-prolyl cis-trans isomerase C | PPIC | | 0.01 | | 0.86 | 1.14 | 0.76 | 6.46 | 6.95 | 1 |
| O43768 | Alpha-endosulfine | ENSA | | 0.00 | | 0.87 | 1.19 | 0.73 | 8.45 | 9.52 | 1 |
| P01040 | Cystatin-A | CSTA | | 0.03 | | 0.87 | 1.21 | 0.72 | 18.07 | 14.85 | 1 |
| Q99592 | Zinc finger and BTB domain-containing protein 18 | ZBTB18 | | 0.04 | | 0.75 | 1.27 | 0.59 | 1.52 | 4.10 | 1 |
